# Data-driven interfacial regulations through molecular additive screening for batteries and electrocatalysis

**DOI:** 10.1039/d6sc04398d

**Published:** 2026-07-17

**Authors:** Guanyu Wang, Shun Zou, Siyuan Lai, Shujie Zhang, Yumo Zhang, Bowen Jiang, Yujie Shi, Guobin Wen, Hongshuai Hou, Bohua Ren, Xiaobo Ji

**Affiliations:** a College of Chemistry and Chemical Engineering, Central South University Changsha 410083 China renbohua@csu.edu.cn; b State Key Laboratory of Chemo and Biosensing, College of Chemistry and Chemical Engineering, Hunan University Changsha 410082 China

## Abstract

Molecular additives are widely employed to regulate electrochemical interfaces, but their rational design remains constrained by fragmented mechanistic understanding and the limited transferability of design principles across batteries and electrocatalysis. This review argues that many additive effects in these systems can be interpreted through three shared interfacial mechanisms: coordination remodeling, interfacial adsorption, and competitive inhibition. In batteries, these mechanisms govern metal deposition, electron-transfer-induced interphase engineering, and the management of reactive intermediates. In electrocatalysis, they underlie activity tuning, pathway steering, and selectivity enhancement through interfacial microenvironment regulation, adsorbate and surface-state regulation, and competing-reaction suppression. Building on this framework, we summarize a data-driven route for additive discovery spanning data mining, predictive modeling, screening, closed-loop optimization, and reasoning-guided and data-enabled exploration. Unlike previous reviews that mainly focus on specific battery chemistries, electrocatalytic microenvironments, or ML-assisted molecular discovery, this review extracts common interfacial mechanisms shared by batteries and electrocatalysis. By organizing molecular additives around shared interfacial primitives, this review identifies descriptor transferability as a key opportunity for data-driven additive design and emphasizes the need for cross-system benchmarks to quantitatively assess generalizability beyond individual chemistries. This framework provides a practical basis for the predictive design of multifunctional additives across electrochemical systems.

## Abstract 2

Data-driven additive discovery is connected to coordination remodeling, interfacial adsorption, and competitive inhibition across batteries and electrocatalysis.

## Graphical abstract

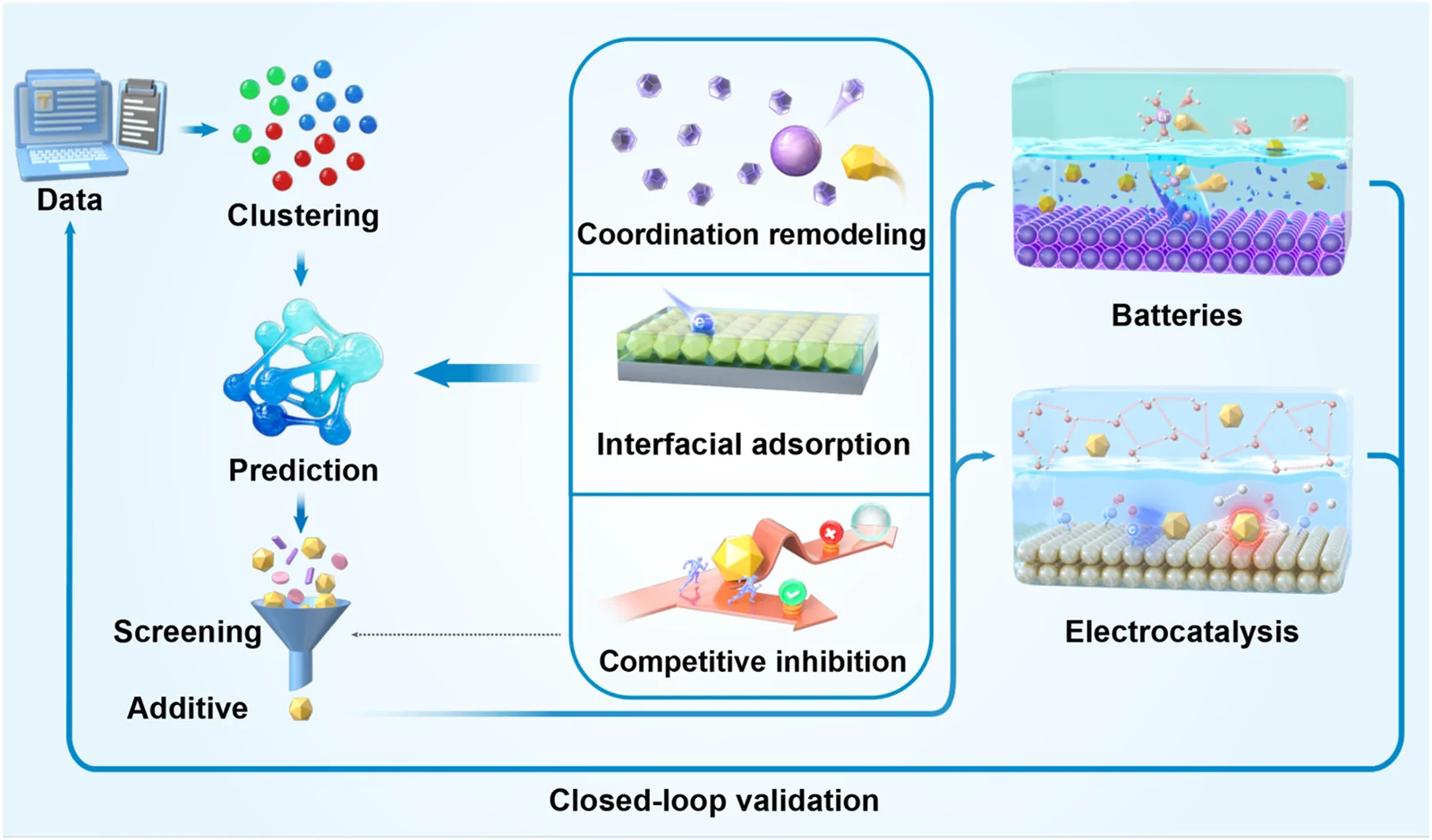

## 1 Introduction

### 1.1 Current landscape of electrochemical molecular additives

Electrochemical technologies support high-energy batteries for transportation and grid storage, as well as electrocatalytic processes that convert renewable electricity into fuels and commodity chemicals.^1–4^ Although electrode materials and device engineering have progressed rapidly,^5^ many key performance limitations still arise from the electrode–electrolyte interface, where the local reaction environment is established and dynamically reshaped during operation.^6–8^ Interfacial charge transfer, solvation structures, and adsorbate populations co-evolve with local electric fields and transport, and together they govern reversibility in batteries as well as activity, selectivity, and durability in electrocatalysis.^9–12^ Accordingly, electrolyte design becomes central to interface control, offering a scalable route to tune interfacial chemistry without modifying the bulk electrode framework.^13–15^

Molecular additives provide an efficient and scalable means of electrolyte engineering, because trace concentrations can reorganize interfacial reaction pathways and translate into device-level gains.^16^ In this review, molecular additives refer to intentionally introduced molecular or ion-associated electrolyte components that regulate electrochemical interfaces without serving as the primary solvent, conductivity-providing supporting salts, bulk electrode components, or catalyst phases. This scope includes neutral organic molecules, functional salt-derived species, specifically adsorbing ions, and soluble or interface-active electrolyte modifiers when their functions involve solvation regulation, interfacial adsorption, reaction-pathway modulation, competitive occupation, or local microenvironment control. This definition separates chemical identity from interfacial function: chemically distinct additive classes may converge on similar mechanistic roles, whereas a single additive can participate in multiple interfacial pathways. In batteries, additives often steer early-stage interfacial chemistry and thereby regulate interphase composition and transport, alleviating impedance growth and stabilizing operation under demanding windows.^17–20^ Additives can further reshape solvation motifs and desolvation kinetics, which govern nucleation behavior, deposition uniformity, and morphological stability during metal plating and stripping.^21–24^ In electrocatalysis, additives and electrolyte modifiers primarily tune the near-surface microenvironment: changes in ion pairing, solvent structuring, and local proton activity alter intermediate coverage and stabilization, redistribute competition among parallel pathways, and suppress parasitic reactions.^25–29^ Viewed through this interfacial lens, additives function as programmable molecular inputs that modulate shared mechanistic primitives across battery and catalytic interfaces.^30^

Despite their promise, additives remain difficult to design in a rational and transferable manner. Their effects are inherently context-dependent, shaped by electrolyte identity and concentration, electrode surface state, operating potential window, temperature, and transport conditions.^31,32^ Consequently, improvements in one metric often incur penalties in others, and mechanistic conclusions drawn under a specific set of conditions may not persist when the formulation or interfacial state is perturbed. This difficulty is compounded by current data-reporting practices: essential metadata are frequently incomplete, unsuccessful formulations are seldom documented, and variations in testing protocols hinder cross-study comparison and model transfer.^33^ These realities underscore the need for an interface-centric, data-structured framework that links mechanism-relevant targets to predictive generalization, enabling additive discovery to progress beyond empirical formulation.^5,34–37^ Previous reviews have provided valuable summaries of electrolyte molecular design in alkali metal batteries, electrocatalytic microenvironment regulation, and machine-learning-assisted battery discovery.^38–40^ These studies have established important system-specific or method-specific design principles, but they generally organize molecular regulation according to application fields or methodological categories. Such organization may obscure the recurring interfacial functions shared by batteries and electrocatalysis. In this review, we therefore reorganize molecular additive effects according to three common mechanistic primitives: coordination remodeling, interfacial adsorption, and competitive inhibition. This cross-system perspective is intended not to replace system-specific design rules, but to connect them through recurring molecular functions that can guide more transferable additive screening. To this end, the following section organizes additive functions through shared mechanistic primitives and corresponding descriptor families, providing a common basis for transferable design rules across batteries and electrocatalysis.

### 1.2 Mechanistic roles and design principles

**Fig. 1:**
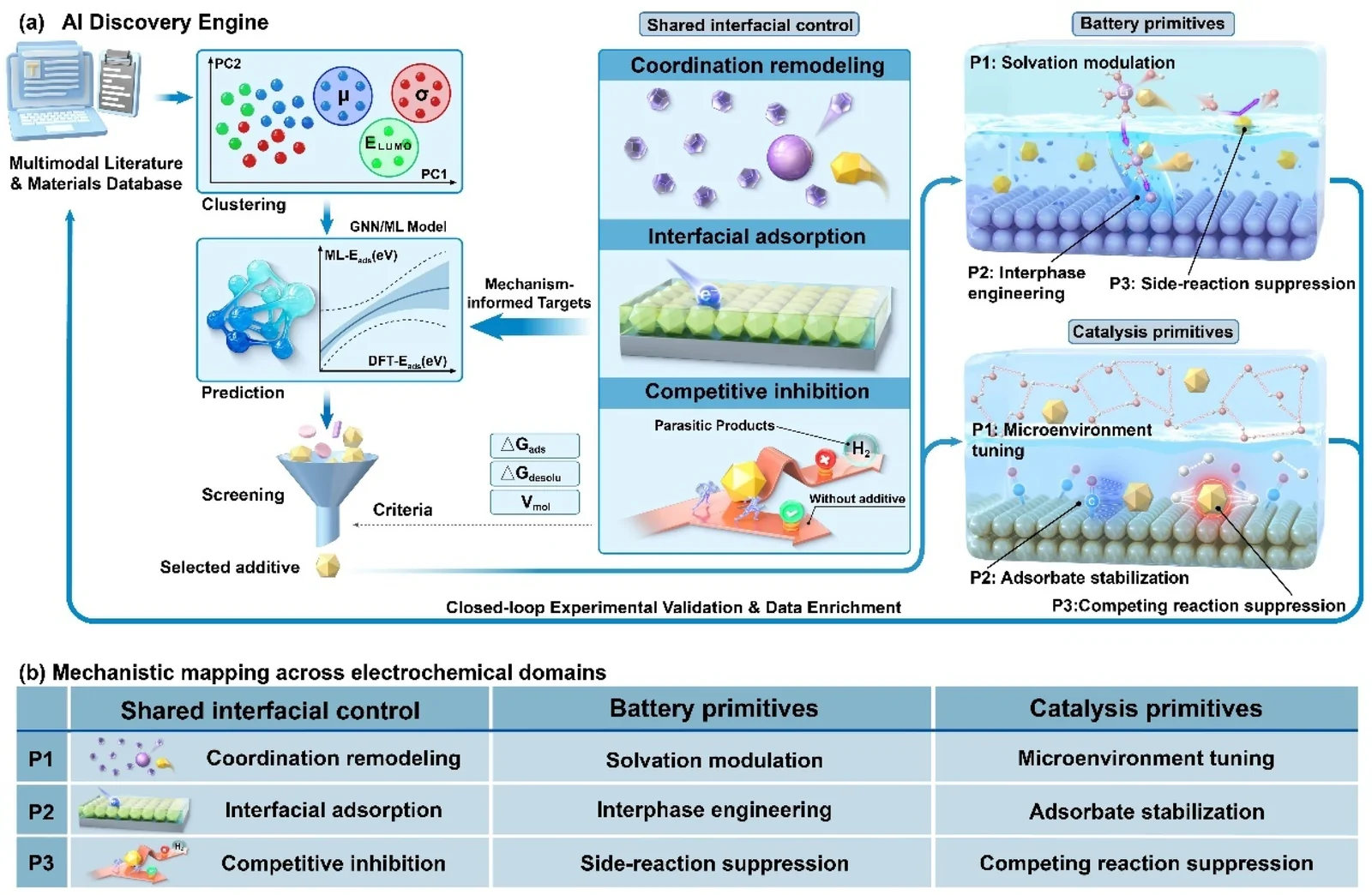
Mechanism-informed AI discovery of multifunctional additives for batteries and electrocatalysis based on shared mechanistic primitives. (a) The AI workflow clusters molecules *via* physicochemical descriptors and screens candidates using thermodynamic and kinetic criteria. The selected additive is visualized in the right panels, functioning through three shared interfacial controls: coordination remodeling, interfacial adsorption, and competitive inhibition. These controls correspond to solvation modulation, interphase engineering, and side-reaction suppression in batteries, and to microenvironment tuning, adsorbate stabilization, and competing reaction suppression in electrocatalysis. (b) Summary of the shared mechanistic primitives and their mapping across battery and catalysis systems. These three primitives are further carried through Sections 2–4 by connecting them to solvation modulation, interphase engineering, side-reaction suppression, adsorbate and surface-state stabilization, and mechanism-informed descriptor construction.

Transferable additive design benefits from a shared description of interfacial processes that can be compared across electrochemical systems. To bridge batteries and electrocatalysis, we organize additive functions into three shared mechanistic primitives: coordination remodeling, interfacial adsorption, and competitive inhibition (Fig. 1). These primitives are not intended to replace conventional electrochemical interpretations of individual examples. Rather, they translate case-specific observations, such as facet-selective adsorption, microenvironment restructuring, or competing-pathway suppression, into a common mechanistic language for connecting battery and catalysis mechanisms within a unified framework for additive discovery. In the following sections, this framework is used as a roadmap rather than only as a graphical summary. In Section 2, coordination remodeling is discussed through solvation modulation, desolvation regulation, and metal-deposition control, while interfacial adsorption and competitive inhibition are further extended to additive-derived interphase engineering and side-reaction suppression in battery systems. In Section 3, the same primitives are translated into electrocatalytic contexts, including local microenvironment regulation, adsorbate and surface-state stabilization, and suppression of competing pathways such as the HER or undesired hydrogenation. In Section 4, these mechanistic primitives are connected to data-driven additive discovery by guiding the selection of mechanism-informed descriptors, such as solvation-related features, adsorption affinity, interfacial stability, and inhibition-related screening targets.

The micro-surface visualizations illustrate the physical expression of these primitives (Fig. 1a). In battery systems, additives regulate local coordination environments to modulate solvation structures, direct interphase formation, and inhibit undesired side-reactions, thereby homogenizing ion flux and stabilizing operation.^8,41–45^ In electrocatalytic systems, additives tune the double-layer microenvironment, stabilize key adsorbates, and suppress competing reactions to improve selectivity and durability.^46,47^ This interface-centric framing also provides a practical basis for descriptor design, where features can be grouped into electronic, solvation, and interfacial families to quantify redox tendency and surface response.

However, translating these primitives into transferable screening rules remains challenging. Additive effects are highly contingent on formulation details and operating windows, often requiring multi-objective trade-offs rather than a single universal metric.^48,49^ This difficulty is compounded by fragmented battery datasets and incomplete metadata, which hinder model transferability.^45^ Under these constraints, machine learning is more useful when trained against mechanism-relevant targets and interfacial representations rather than device-level endpoints alone.^50^

Consequently, the AI workflow shown in Fig. 1a should be built on consistent validation standards.^51,52^ The process begins with reaction-relevant knowledge organization, where clustering links additive families to recurring interfacial modes of action.^53,54^ Physics-based and learnable descriptors then map the molecular structure to interfacial processes, enabling predictions that are comparable across systems.^55^ Models that integrate interpretability and uncertainty quantification further support screening by flagging unreliable extrapolations,^56^ but their reliability still depends strongly on data governance and language model assisted extraction to ensure coverage and consistency.^57–59^ Although data-efficient optimization can reduce exploration costs, robust generalization remains limited by mechanistic fidelity.^60^

Existing reviews often treat battery mechanisms and catalytic microenvironments in isolation.^15,61–65^ By contrast, this work connects these domains through shared mechanistic primitives aligned with descriptor families, thereby clarifying the requirements for transferable, data-driven additive design. At the same time, these primitives should be understood as a mechanism-guided organizing framework rather than a quantitatively validated universal descriptor set; their predictive transferability must ultimately be assessed through standardized cross-system datasets and validation protocols.

## 2 Molecular additives in electrochemical energy storage systems

### 2.1 Solvation and interfacial regulation of metal deposition

**Fig. 2:**
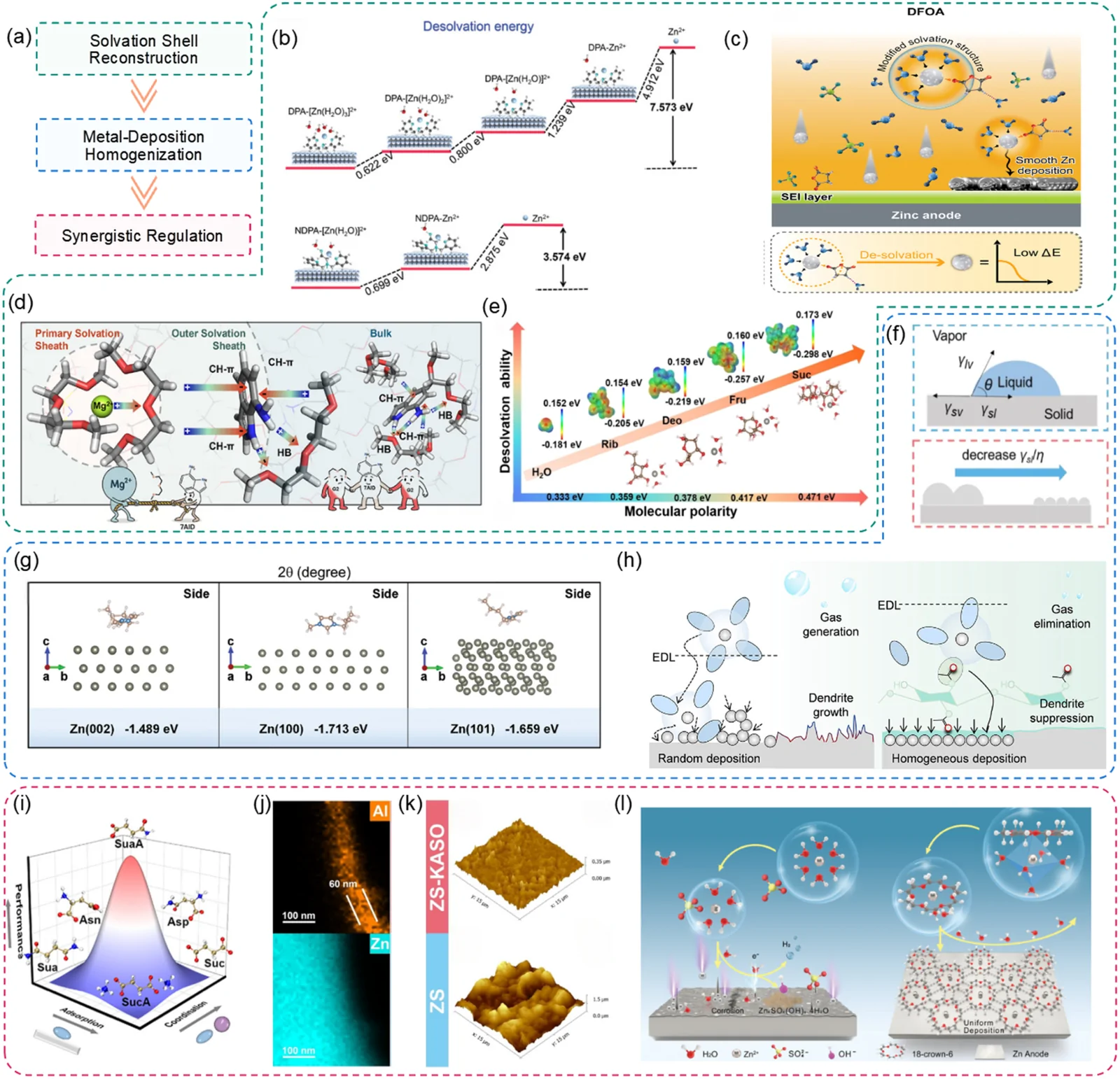
Solvation and interfacial regulation of metal deposition. (a) Schematic overview of the hierarchical logic flow, from single-target deposition optimization to synergistic strategies. (b) Schematic of Zn^2+^ five-coordinated by one H_2_O molecule and one NDPA molecule, resulting in a lower desolvation energy compared to other systems.^66^ Copyright 2025 Wiley. (c) Schematic illustration of additive-induced Zn^2+^ solvation regulation, reduced desolvation barrier, SEI formation, and smooth Zn deposition.^67^ Copyright 2025 Wiley. (d) Schematic illustration of 7AID regulating Mg^2+^ desolvation through the outer solvation shell and bulk phase interactions with solvent molecules.^68^ Copyright 2025 Wiley. (e) Schematic diagram of the relationship between electrostatic potential and desolvation capability for five saccharide molecules.^69^ Copyright 2023 Wiley. (f) Schematic illustration of initial nucleation behavior that varies with the ratio of *γ*_sl_/*η*.^70^ Copyright 2023 Wiley. (g) Schematic of adsorption modes and adsorption energies of BMIm^+^ ions on different crystal planes of Zn metal.^71^ Copyright 2022 Wiley. (h) Schematic comparison of the Na^+^ coordination environment and deposition behavior in electrolytes with a CLA additive *versus* the base electrolyte.^72^ Copyright 2025 American Chemical Society. (i) Volcano relationship between adsorption coordination interactions and electrochemical performance for six C_4_ chain molecules.^73^ Copyright 2025 Elsevier. (j) Cross-sectional EDS mapping reveals the formation of a Zn–Al alloy interface on the Zn anode after cycling in the electrolyte containing the KAl(SO_4_)_2_ additive, resulting in (k) the improved Zn surface morphology compared to that when cycled in the base electrolyte.^74^ Copyright 2024 Wiley. (l) Schematic comparison of interfacial chemistry on zinc anodes in the base electrolyte *versus* the electrolyte containing the 18-crown-6 additive.^75^ Copyright 2025 Wiley.

Before metal ions reach the electrode surface to undergo charge transfer, they must first navigate the physicochemical evolution of the coordination environment within the bulk electrolyte and at the interface. Metal electrodeposition proceeds *via* a sequential cascade and is frequently impeded by kinetic bottlenecks, particularly high desolvation penalties and stochastic nucleation that precipitate dendritic failure. Molecular additives intervene by remodeling the coordination environment, thereby modulating solvation structures and desolvation kinetics. Accordingly, this section is organized into three levels of discussion: reconstruction of the solvation shell to reduce desolvation barriers, regulation of nucleation and ion flux to homogenize metal deposition, and synergistic strategies that balance interfacial stability with reaction kinetics (Fig. 2a).

#### 2.1.1 Solvation shell reconstruction

Rational reconstruction of the primary solvation shell is fundamental to optimizing interfacial kinetics. Acting as competitive ligands, functional additives displace reactive solvents to attenuate desolvation penalties. This strategy is pivotal in aqueous zinc-ion batteries, where water-dominated solvation drives parasitic hydrogen evolution. High-affinity polar additives competitively exclude active water from the primary sheath. This substitution delivers a dual benefit: thermodynamically reducing local water activity and kinetically disrupting the bulk hydrogen-bond network, thereby suppressing proton transport and ensuring highly reversible deposition.

Additives with competitive coordination capabilities generally exhibit higher affinity for metal ions than solvents. To minimize the desolvation penalty, however, these molecules must retain appropriate solvation affinity, enabling solvent-mediated ligand dissociation. *N*,*N*-Di-(2-picolyl) ethylenediamine (NDPA) exemplifies this mechanism. Its multidentate architecture imposes significant steric bulk, driving the Zn^2+^ primary shell from a stable hexa-coordinated state to a metastable penta-coordinated geometry.^66^ This lower coordination number directly reduces the desolvation energy barrier (Fig. 2b). Concurrently, amino motifs facilitate ligand dissociation *via* hydrogen-bonding interactions with bulk water. These synergistic effects accelerate interfacial kinetics, ensuring rapid and reversible metal deposition.

A parallel strategy is observed with succinic anhydride, which simultaneously modulates the bulk solvation environment and the electrode interface. Hazoor *et al.*^67^ demonstrated that this molecule competitively displaces water within the primary coordination sphere, effectively attenuating zinc–water interaction strength. This substitution lowers the desolvation energy barrier (Fig. 2c). Concurrently, the preferential adsorption of succinate anions directs the *in situ* formation of a robust SEI layer, thereby coupling kinetic acceleration with interfacial passivation.

Expanding the regulatory scope beyond direct cation coordination, additive-solvent interactions offer a potent avenue for “second-sphere” engineering. In magnesium battery systems, the amine-based agent 7AID exemplifies this *via* a competitive solvent-additive interaction mechanism.^68^ Rather than displacing solvents from the primary sheath, 7AID exerts an external molecular pull that destabilizes the coordination environment, effectively lowering the energetic barrier for desolvation (Fig. 2d). Concurrently, interactions between the additive and bulk solvent enhance molecular mobility and entropically constrain the availability of coordinating species. This underscores a critical design hierarchy: optimizing interfacial kinetics requires modulating the outer solvation shell and bulk-phase dynamics alongside the primary coordination sphere.

Transitioning from empirical observation to quantifiable structure–activity relationships, Zhou *et al.*^69^ identified electrostatic polarity as a decisive descriptor for saccharide additives. A positive correlation links molecular polarity with interfacial regulation (Fig. 2e). Highly polar candidates, exemplified by sucrose, exhibit superior capability to competitively displace water and reconstruct the solvation environment. By enabling the formation of compact adsorption layers, this polarity-driven principle provides a sound basis for the rational screening of non-sacrificial additive candidates with enhanced kinetic regulation capabilities.

Lowering desolvation barriers alone does not guarantee uniform deposition. Once metal ions approach the surface, interfacial adsorption and nucleation control become the next determinants of deposition morphology.

#### 2.1.2 Metal-deposition homogenization

Beyond solvation dynamics, functional additives directly govern deposition morphology by modulating interfacial adsorption and nucleation. From a kinetic perspective, these agents lower nucleation barriers to promote fine, dense growth, while selective adsorption dictates crystallographic orientation. By leveraging mechanisms ranging from electrostatic shielding to facet-selective growth, additives effectively guide uniform ion flux. Precise interfacial modulation plays a critical role in homogenizing metal deposition, establishing a pathway toward dendrite-free reversibility.

Chemisorption-mediated regulation effectively lowers nucleation barriers. Jiang *et al.*^70^ demonstrated that cysteine modulates interfacial energetics *via* thiol-anchored adsorption. This stable layer reduces solid–liquid interfacial energy and improves wettability. Under classical nucleation theory, a lower overpotential decreases the critical radius (*r*_crit_) for nucleation, driving a definitive transition from sparse, island-like grains to dense, fine nuclei (Fig. 2f). By masking high-energy sites, this mechanism homogenizes local reduction rates to ensure uniform deposition morphology.

Functional additives can further homogenize deposition morphology by inducing facet-selective metal growth. Zhang *et al.*^71^ introduced the 1-butyl-3-methylimidazolium cation (BMIm^+^) as an electrolyte additive for Zn-based batteries, which promotes preferred growth along the Zn (002) plane and thus suppresses dendritic growth. BMIm^+^ selectively adsorbs on the more reactive Zn (100) and Zn (101) facets (Fig. 2g), inhibiting Zn^2+^ deposition on these planes and thereby redirecting growth toward the thermodynamically more stable (002) facet. This facet-regulation strategy enables directional deposition and improved morphological stability of the Zn anode. Within the primitive framework, BMIm^+^ can therefore be assigned to interfacial adsorption coupled with competitive blocking: preferential adsorption on Zn (100)/(101) facets suppresses local Zn^2+^ reduction on high-reactivity planes and redirects growth toward the Zn (002) orientation.

Targeting the Inner Helmholtz Plane (IHP) offers precise control over the electric double layer (EDL). Zhou *et al.*^72^ employed cellulose acetate to reconstruct this critical interface in sodium metal batteries. The additive preferentially adsorbs at the IHP while competitively engaging in solvation. This dual action attenuates the desolvation penalty and reshapes the local electric field distribution (Fig. 2h). Consequently, the reconstructed EDL homogenizes Na^+^ flux, effectively coupling accelerated kinetics with robust dendrite suppression.

Viewed together, these studies show that additives homogenize metal deposition by redistributing ion flux, lowering nucleation barriers, and steering crystallographic growth through interfacial adsorption. However, stronger surface regulation does not always translate directly into faster deposition, because adsorption-induced homogenization can also increase charge-transfer resistance. This trade-off motivates the synergistic strategies discussed next.

#### 2.1.3 Synergistic strategies enhancing interfacial stability and reaction kinetics

Metal electrodeposition is intrinsically constrained by a “nucleation-charge transfer paradox.” Strong interfacial adsorption promotes uniform nucleation but frequently imposes a kinetic penalty by increasing charge-transfer resistance, whereas rapid kinetics without regulation risks uncontrolled, coarse growth. Resolving this trade-off requires synergistic strategies that integrate solvation control, interfacial adsorption, and field regulation. By balancing thermodynamic stability against kinetic acceleration, these strategies enable uniform nucleation while sustaining high-rate, orderly deposition.

To quantitatively rationalize adsorption-coordination synergy, Duan *et al.*^73^ proposed a volcano-type model to describe how the relative strengths of interfacial adsorption and Zn^2+^ coordination jointly determine deposition morphology. By comparing six organic molecules with identical carbon-chain lengths but different functional groups, they identified a clear volcano relationship between adsorption strength and coordination ability (Fig. 2i). Succinamic acid (SuaA), exhibiting moderate adsorption and coordination, resides near the volcano apex, where a balanced interaction favors three-dimensional progressive nucleation and controlled Zn growth. This model provides a quantitative framework for optimizing the adsorption-coordination balance in additive design.

Beyond organic additives, ionic species can also realize analogous adsorption-coordination synergy. He *et al.*^74^ demonstrated that Al^3+^ simultaneously modulates Zn^2+^ solvation and deposition selectivity. Specifically, Al^3+^ competitively coordinates in the electrolyte to reduce the population of active water molecules around Zn^2+^, while preferential electrodeposition of Al^3+^ leads to the formation of a Zn–Al alloy interphase (Fig. 2j). This alloy layer homogenizes the interfacial electric-field distribution and lowers the nucleation barrier, thus enabling comprehensive optimization of Zn deposition behavior (Fig. 2k).

Complementary to surface networks, molecular-scale ion channels offer robust flux regulation. Wu *et al.*^75^ utilized 18-crown-6 to construct specific transport pathways *via* its cavity structure. In the bulk, this enhances cation transference; at the interface, strong adsorption creates a dynamic shielding layer that guides nucleation. The confined ring topology compels hydrated cations to undergo partial desolvation (Fig. 2l). This coupled modulation of bulk transport and interfacial energetics lowers reaction barriers, enabling rapid, dense zinc nucleation.

In this sense, the “nucleation-charge transfer paradox” reflects the intrinsic tension among several coupled requirements for stable metal deposition. Solvation-shell reconstruction can lower the desolvation barrier and improve interfacial kinetics, but overly strong metal-additive coordination may slow ligand dissociation or ion migration. Ion-flux homogenization and facet-selective adsorption can promote uniform nucleation and directional growth, but excessive surface adsorption may block active interfacial sites and increase charge-transfer resistance. Therefore, the underlying design principle is not the maximization of any single interaction, but the balanced regulation of coordination strength, interfacial adsorption, and ion-flux distribution across the electrolyte-interface continuum. This balanced view is consistent with recent solvation-centric electrolyte design principles, in which solvation strength, ion transport, interfacial decomposition, and interphase stability are treated as coupled design variables rather than independent optimization targets.^76^

### 2.2 Electron-transfer-induced interphase engineering

**Fig. 3:**
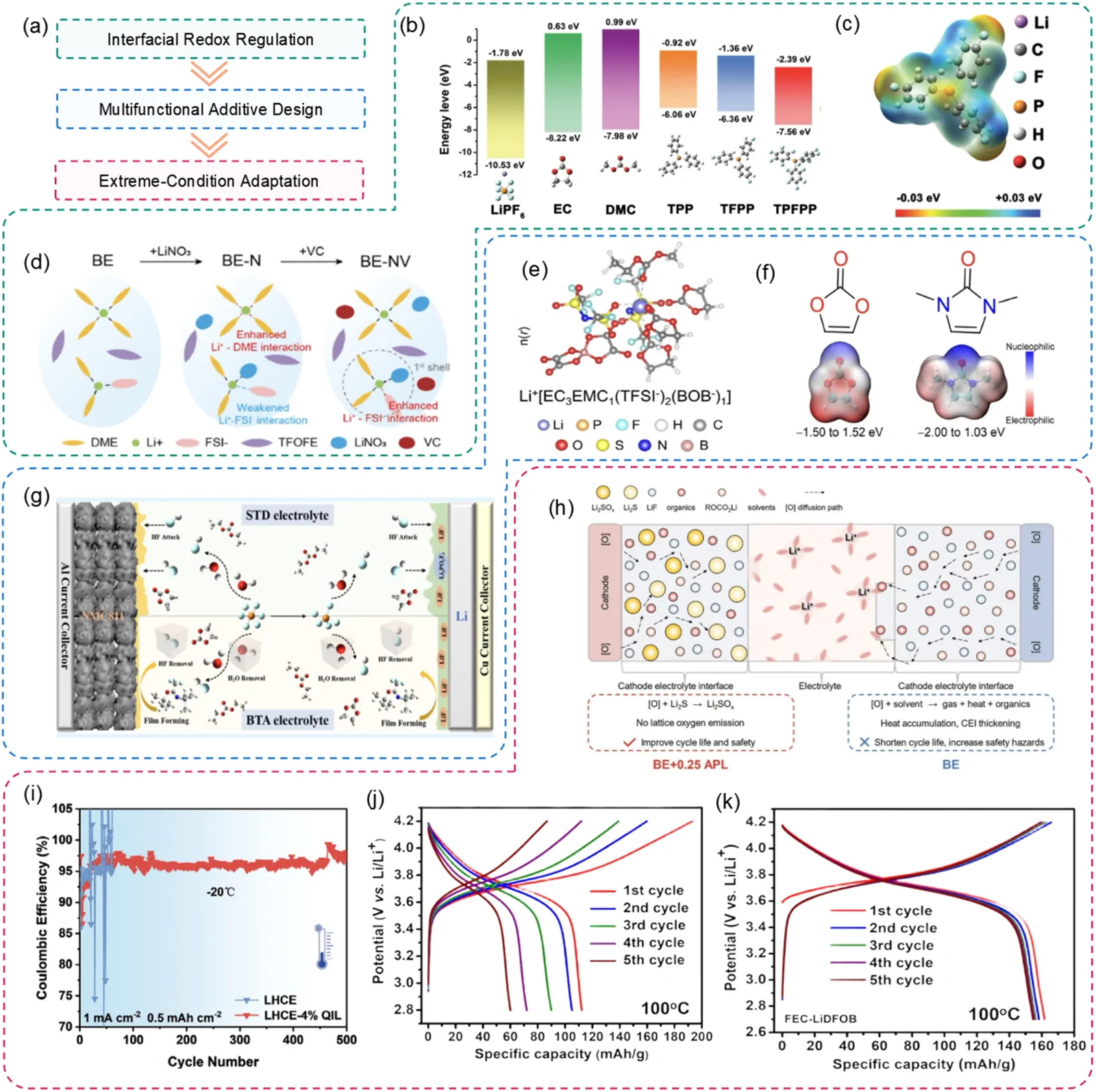
Electron-transfer-induced interphase engineering. (a) Schematic overview of the hierarchical logic flow, progressing from fundamental redox potential regulation based on frontier orbitals, synergistic functional design for multi-component interphases, and extreme environment adaptation ensuring robust operation under thermal and electrochemical stress. (b) Theoretical calculation of LUMO and HOMO values of LiPF_6_, solvents and additives.^77^ Copyright 2022 Wiley (c). The ESP charge distribution of TFPP molecules.^77^ Copyright 2022 Wiley (d). The schematic illustration for different interactions of Li^+^ with the DME solvent and FSI^−^ anions within solvation structures in the three electrolytes.^78^ Copyright 2024 American Chemical Society. (e) Representative solvation structures of BTB electrolytes.^82^ Copyright 2025 Chemistry Europe. (f) Molecular structures and electrostatic potentials of the VC and DMIO.^83^ Copyright 2024 American Chemical Society. (g) Illustration of the multi-functional BTA additive facilitating the formation of LiF-rich, Si- and N-containing solid electrolyte interphase films on both Li metal anode and LiNi_0.8_Mn_0.1_Co_0.1_O_2_ (NMC811) cathode surfaces.^84^ Copyright 2021 American Chemical Society. (h) Schematic of capturing active oxygen species by the CEI formed in BE + 0.25 APL.^86^ Copyright 2024 American Chemical Society. (i) The CE comparison of Li/Cu cells at −20 °C in LHCE and LHCE-4% QIL.^87^ Copyright 2024 Wiley. (j) The galvanostatic charge–discharge profile of NMC333 at 100 °C in ionic liquid electrolytes without additives.^88^ Copyright 2022 American Chemical Society. (k) 2 wt% FEC and 2 wt% LiDFOB.^88^ Copyright 2022 American Chemical Society.

Following desolvation, interfacial electron transfer initiates solid electrolyte interphase (SEI) formation, thereby introducing an additional level of additive control beyond solvation and deposition behavior. The physicochemical architecture of the SEI dictates the reversibility and kinetics of high-energy batteries. Constructing an interphase that effectively decouples electron transport from ion migration therefore requires precise molecular engineering. Mechanistic understanding has now moved beyond the traditional “sacrificial” view toward a more rigorous framework of electron-transfer control. This hierarchical logic progresses from fundamental redox regulation *via* frontier orbitals to multifunctional additive design and, ultimately, adaptation under extreme operating conditions (Fig. 3a).

#### 2.2.1 Interfacial redox regulation

Departing from the traditional “sacrificial” paradigm, Wu *et al.*^77^ decoded the electronic origin of additive efficacy using fluorinated phenylphosphines. Increased fluorination depresses LUMO levels, establishing a thermodynamic gradient that compels preferential reduction (Fig. 3b). However, thermodynamic priority requires kinetic reinforcement. A critical “kinetic takeover” occurs as low bond dissociation energies drive the rapid polymerization of intermediates (Fig. 3c). This fast pathway kinetically supersedes disordered solvent breakdown, constructing a dense LiF-rich shield that effectively arrests parasitic interfacial reactions.

Manipulating interaction strength within the primary solvation shell can precisely govern film formation rates. As shown by Park *et al.*,^78^ specific additives reinforce cation–anion coordination, elevating the dissociation energy barrier (Fig. 3d). This induced “kinetic deceleration” affords decomposition products sufficient time to crystallize into a thin, uniform inorganic layer. Conversely, additives that weaken this coordination trigger rapid, disordered solvent breakdown, yielding thick, organic-rich films. Consequently, optimizing compatibility between additives and the solvation structure is essential to balance ion transport with interfacial crystallinity.

More broadly, these findings mark a conceptual leap, transforming the view of additives from passive “sacrificial agents” to active “redox regulators.” Whether by modulating frontier orbital energies to dictate thermodynamic priority or manipulating solvation coordination to induce “kinetic deceleration,” additives now regulate the entire interfacial lifecycle. This precise control over electron-transfer pathways effectively decouples interfacial stability from bulk transport, establishing the foundational logic for the multi-component and multifunctional design strategies discussed next. Taken together, frontier-orbital and bond-dissociation regulation define the initial chemical trigger for additive-derived interphase formation. HOMO/LUMO-related redox tendencies provide an initial guideline for whether additives, solvents, or salts preferentially participate in early SEI/CEI formation, although the actual interfacial reaction pathway is further shaped by the local coordination and electrode environment.^79^

#### 2.2.2 Evolution from single-function to multifunctional additive design

Practical electrode instability rarely originates from a single interfacial process. In high-energy-density batteries, increased operating voltage and high-capacity electrode materials simultaneously challenge electrolyte stability, electrode surface chemistry, impurity tolerance, and interphase transport.^80^ Historically, electrolyte additive design followed a reductionist paradigm, in which discrete molecules were used to address specific interfacial deficits such as solvent decomposition or metal dissolution. However, monofunctional strategies often prove insufficient in high-energy cells, where anodic and cathodic instabilities are intrinsically coupled. The field has therefore shifted toward combinatorial synergy and integrated multifunctionality to address the full complexity of the electrochemical cell.

To illustrate this limitation, Li *et al.*^81^ employed sodium lactate to mitigate Mn dissolution in aqueous Zn-ion batteries. The preferential adsorption of lactate anions effectively constructs a protective cathode interphase, which physically blocks ion loss and suppresses irreversible Jahn–Teller distortions. However, while this strategy significantly enhances cathode stability, its efficacy remains strictly localized. The additive lacks the capacity to simultaneously modulate zinc anode deposition or regulate the bulk solvation structure. This case exemplifies the inherent limitation of monofunctional design, where exceptional protection at one interface often fails to address the coupled instabilities of the full electrochemical cell.

To address distinct anode and cathode instabilities, the field has advanced toward combinatorial strategies. For example, Chu *et al.*^82^ exemplified this with a dual-anion protocol for high-voltage Li-metal batteries. Strong-coordinating anions (TFSI/BOB) competitively displace solvents, reshaping the primary sheath into an anion-dominated architecture (Fig. 3e). This configuration drives the preferential formation of a conductive, inorganic-rich interphase. By concurrently suppressing high-voltage oxidation and transition metal dissolution, the design validates that precise molecular pairing induces a synergistic effect, where collective protection exceeds the sum of individual components.

A more advanced strategy in additive engineering is the development of integrated multifunctional molecular scaffolds. Wang *et al.*^83^ demonstrated this with DMIO, a vinylene carbonate derivative. Strategic *N*-substitution elevates the HOMO level to induce preferential cathodic oxidation, while the preserved C=C bond enables anodic polymerization (Fig. 3f). Consequently, a single DMIO molecule directs the simultaneous construction of a nitrogen-rich CEI and a robust SEI. Beyond this bipolar protection, its intrinsic Lewis basicity scavenges acidic impurities. This “single-molecule, multi-target” paradigm effectively decouples interfacial stability from electrolyte complexity, streamlining the path to high-voltage durability.

Parallel to electronic modulation, Liu *et al.*^84^ proposed BTA as a comprehensive stabilizer. Its molecular architecture strategically couples hydrolyzable silyl groups with a fluorinated backbone. The Si–N and Si–O motifs function as potent Lewis bases, aggressively scavenging moisture and HF to arrest bulk electrolyte degradation, while the trifluoromethyl moiety serves as a critical fluorine donor. Through this bipolar mechanism, BTA constructs stable Si/N-doped shields on both electrodes (Fig. 3g). By consolidating impurity scavenging with interfacial passivation, this design enables Li‖NMC811 cells to sustain robust cycling even under rigorous conditions.

At a broader level, electrolyte additive design has evolved from single-function molecules to synergistic combinations and integrated multifunctionality. The value of these strategies is most rigorously tested under extreme operating conditions, where conventional interphases are pushed to their limits.

#### 2.2.3 Additive design under extreme conditions

The robustness of additive-derived interphases becomes particularly important under extreme operating conditions. High voltage, low temperature, and high temperature amplify different failure modes, including oxidative electrolyte decomposition, sluggish interfacial transport, dendritic metal growth, and thermally accelerated structural degradation. Under such conditions, additive-derived SEI/CEI layers must remain chemically protective while still supporting ion transport, making extreme operation a practical stress test for interphase robustness.^85^ These conditions do not merely amplify conventional degradation, but reshape the interfacial reaction landscape and provide a stringent validation platform for advanced molecular engineering strategies.

High-voltage operation triggers lattice oxygen release and severe solvent decomposition. Wu *et al.*^86^ addressed this by utilizing 4-aminothiophenol to engineer a gradient interface on Ni-rich cathodes. The *in situ* conversion of sulfide species to sulfates actively captures reactive lattice oxygen, thereby arresting the oxidative cascade (Fig. 3h). This scavenging mechanism preserves structural integrity and suppresses transition metal dissolution, enabling stable electrochemical performance at ultra-high voltages (4.8 V).

To overcome the kinetic sluggishness at low temperatures, He *et al.*^87^ addressed the solubility limit of the fast-conducting nitride precursor lithium nitrate in carbonate electrolytes. By engineering a crown ether-based quasi-ionic liquid additive, they leveraged strong chelation to dissolve nitrate species. This facilitates the *in situ* construction of a highly conductive, nitride-rich interphase that lowers the activation energy for interfacial transport. Consequently, highly reversible lithium deposition is achieved at −20 °C (Fig. 3i), offering a precise solution to the arrested dynamics of cryogenic batteries.

High-temperature operation acts as a thermodynamic stress test, accelerating cathode phase transitions. To counter this, Nagarajan *et al.*^88^ employed a LiDFOB/FEC combination to engineer a gradient interphase. Depth profiling identifies a “thermal shield” architecture where stable outer boron–oxygen species protect an inner fluoride-rich layer. This composition effectively arrests the detrimental layered-to-spinel transformation at 100 °C. As a result, the modified system maintains stable voltage plateaus, contrasting sharply with the severe polarization observed in the unprotected control (Fig. 3j and k).

From this perspective, extreme operating conditions expose the limits of conventional electrolytes and highlight the need for more targeted additive design. Beyond maintaining interphase integrity at harsh voltages and temperatures, practical batteries must also regulate soluble intermediates and corrosive species generated during cycling.

### 2.3 Side-reaction suppression and management of reactive intermediates

**Fig. 4:**
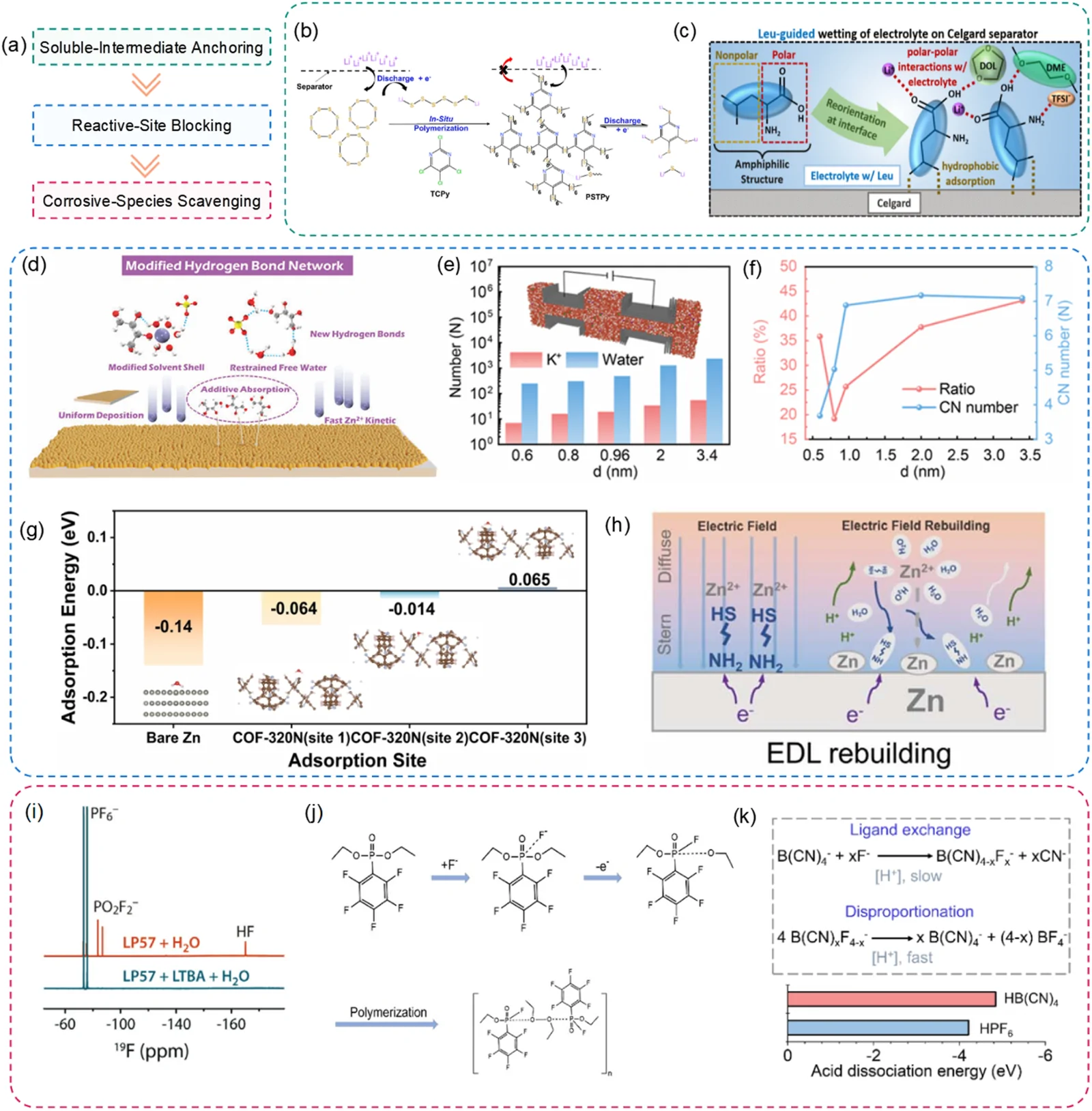
Management of reactive intermediates and suppression of parasitic pathways. (a) Overview of the main mechanisms by which molecular additives suppress side reactions in batteries. (b) Schematic of the electrochemical polymerization mechanism of TCPy during discharge.^90^ Copyright 2025 Elsevier. (c) Schematic illustrating the mechanism of separator interface regulation *via* coordinative anchoring by l-leucine.^91^ Copyright 2025 Elsevier. (d) Schematic illustration of erythritol-mediated regulation of the Zn^2+^ solvation shell, hydrogen-bond network, additive adsorption, and Zn deposition behavior.^92^ Copyright 2024 Wiley. (e) Simulated numbers of K^+^ and water molecules in bioinspired ion channels with different pore sizes.^93^ Copyright 2025 American Chemical Society. (f) Water-to-K^+^ ratio and hydration coordination number as a function of pore size, illustrating pore-size-dependent desolvation regulation.^93^ Copyright 2025 American Chemical Society. (g) Adsorption energies of H_2_O molecules on bare Zn and different COF-320N adsorption sites, showing weakened water–surface interaction after COF membrane regulation.^94^ Copyright 2025 Wiley. (h) Schematic illustration of Cy-H-mediated electric-double-layer rebuilding for suppressing parasitic reactions and enabling targeted by-product elimination at the Zn anode interface.^98^ Copyright 2025 Royal Society of Chemistry. (i) Comparative analysis of ^19^F NMR chemical shifts for PF_6_^−^ and PO_2_F_2_^−^ anions in electrolytes regulated by LTBA.^99^ Copyright 2024 Wiley. (j) Schematic representation of the proposed mechanism for electrochemical reductive decomposition of FPOP additives.^100^ Copyright 2024 Elsevier. (k) HF regulation mechanism mediated by TCB: ligand exchange and disproportionation pathways and acid dissociation energy analysis.^102^ Copyright 2025 Elsevier.

Beyond morphological control, managing soluble and reactive intermediates constitutes a critical bottleneck, particularly in sulfur and aqueous chemistries. The uncontrolled migration of these species precipitates parasitic cascades and continuous active material loss. Functional additives intervene by coordinating targeted interactions within the electrolyte and at interfaces. This regulatory hierarchy categorizes mechanisms into three distinct strategies: anchoring soluble intermediates to restrict mobility, blocking reactive sites to shield interfaces, and scavenging corrosive species to neutralize threats before they initiate degradation (Fig. 4a).

These additive functions can be distinguished by the regulated species and the level of mechanistic evidence required for assignment. Anchoring mainly involves the immobilization of soluble intermediates, typically polysulfides, through coordination, covalent, electrostatic, or host–guest interactions. Catalytic conversion further requires evidence that the additive accelerates the redox transformation of captured intermediates, rather than merely retaining them at the interface. Blocking refers to interfacial shielding or passivation that suppresses the access of reactive species to active electrode sites, whereas scavenging denotes chemical consumption, neutralization, or targeted elimination of corrosive species and accumulated by-products. Because these functions often coexist in practical additive systems, dominant-function assignment provides a useful basis for comparing design principles across Li–S, aqueous, and high-voltage battery chemistries.

#### 2.3.1 Polysulfide-shuttle suppression in lithium–sulfur batteries

The diffusion and shuttling of soluble polysulfide species give rise to side reactions on the lithium surface, representing a major issue in lithium sulfur batteries. Recent studies demonstrate that molecular additives can effectively suppress the polysulfide shuttle through chemical anchoring, often synergistically coupled with catalytic conversion or interfacial blocking, thereby stabilizing redox pathways and improving reaction reversibility. In Li–S chemistry, this distinction separates immobilization-dominated strategies from conversion-promoting strategies: restricted polysulfide dissolution or migration supports an anchoring assignment, whereas catalytic conversion requires additional kinetic evidence, such as accelerated polysulfide redox reactions or reduced conversion barriers between soluble Li_2_S_*x*_ and insoluble Li_2_S/Li_2_S_2_.

Moving beyond localized anchoring, Zhao *et al.*^89^ introduced a cross-location synergy paradigm using ZnI_2_. This additive orchestrates regulation across the entire electrochemical pathway. At the cathode, *in situ* generated ZnS chemically anchors and catalytically converts polysulfides. Simultaneously, a Li–Zn alloy interphase at the anode effectively blocks parasitic reduction. Coupled with solvation modulation, this holistic approach validates that regulating the full cell ecosystem yields stability far superior to single-point interventions.

Using a stimulus-responsive design, Ma *et al.*^90^ introduced tetrachloropyrimidine (TCPy). Unlike passive barriers, TCPy triggers an *in situ* interfacial reconstruction upon discharge. Rapid nucleophilic substitution between chlorinated sites and polysulfide anions drives the formation of an insoluble, crosslinked polymer film (PSTPy) (Fig. 4b). This covalent C–S framework serves as a dual barrier, simultaneously enabling chemical immobilization and physical blocking. Reinforced by catalytic nitrogen sites that promote conversion, this “covalent trapping” pathway effectively arrests polysulfide dissolution and diffusion at the source.

Conversely, moderate anchoring offers a dynamic pathway to couple capture with conversion. Zhong *et al.*^91^ utilized l-leucine to establish a “gentle” coordination regime. Carbonyl oxygen sites form intermediate-strength Li–O bonds that capture polysulfides without kinetically trapping them (Fig. 4c). Crucially, this interaction modulates the electronic structure, lowering the activation barrier for the reduction of Li_2_S_*x*_ to solid Li_2_S. By balancing adsorption strength with catalytic turnover, this dynamic interplay effectively suppresses the shuttle effect while maintaining rapid interfacial kinetics.

Taken as a whole, effective additive design in Li–S batteries increasingly relies on coordinated regulation across multiple interfaces rather than on a single mechanism. Rather than relying on isolated anchoring, advanced strategies integrate chemical immobilization, catalytic turnover, and interfacial reconstruction. This comprehensive regulation scheme governs polysulfide behavior across the entire cell environment—the cathode, anode, and bulk electrolyte. Accordingly, effective shuttle suppression requires coordinated control over both thermodynamic stability and kinetic pathways, providing a useful framework for designing more durable high-energy sulfur chemistries.

#### 2.3.2 Parasitic-reaction suppression in aqueous batteries

While Li–S batteries are primarily challenged by the migration of soluble redox intermediates, aqueous batteries are more strongly limited by parasitic reactions driven by active water. Suppressing these reactions requires coordinated regulation of the solvation sheath, hydrogen-bond network, and electrode interface, which is the focus of the following aqueous-battery examples.

In aqueous batteries, blocking is more closely associated with active-water exclusion and suppression of water-derived parasitic reactions than with immobilization of soluble redox intermediates. For example, erythritol additives regulate the Zn^2+^ solvation structure, reconstruct the hydrogen-bond network, and preferentially adsorb on the Zn surface to form a dynamic protective layer, thereby coupling active-water suppression with improved Zn^2+^ transport and uniform deposition (Fig. 4d).^92^

Beyond molecular additives, bioinspired interfacial regulators further extend this water-regulation logic in aqueous systems. Deng *et al.* designed subnanometer ion channels that promote hydrated-ion desolvation and reduce interfacial water decomposition in nonconcentrated aqueous electrolytes. The pore-size-dependent ion and water distributions, together with the decreased hydration coordination environment, directly illustrate that desolvation-assisted water regulation can suppress parasitic water reactivity (Fig. 4e and f).^93^ In a related membrane-based strategy, Zhang *et al.* developed zincophilic COF membranes that regulate free and solvated water at the anode–electrolyte interphase, accelerate Zn^2+^ transport/desolvation, and reduce direct contact between water and the Zn surface. The weakened adsorption of H_2_O on COF-320N relative to bare Zn further supports the role of interfacial shielding in mitigating water-induced side reactions (Fig. 4g).^94^ Together, these studies broaden the evidence basis for HER suppression from molecular water exclusion to desolvation-assisted water regulation and interfacial shielding.

This logic is reflected in molecular additive examples that simultaneously regulate solvation, water activity, and interfacial contact. Liu *et al.*^95^ employed tripropylene glycol (TG) as a bifunctional modulator. TG competitively displaces water molecules from the primary Zn^2+^ solvation shell, significantly reducing the amount of electrochemically active water. Concurrently, it reorganizes the bulk hydrogen-bond network and establishes a dynamic protective layer. This multi-level defense, which couples thermodynamic water exclusion with physical shielding, effectively suppresses the HER and secures highly reversible anode operation. Bai *et al.*^96^ revealed a triple cooperative mechanism using Azithromycin (Azi). Azi coordinates Zn^2+^*via* N/OH functionalities to displace active water. Beyond this reconstruction, it forms a compact adsorption layer that physically screens HER active sites. Finally, strong Azi–water interactions disrupt the global hydrogen bond network. This synergistic regulation simultaneously constrains interfacial water availability and dampens bulk water reactivity to comprehensively arrest parasitic evolution.

Homogenizing deposition serves as an often-overlooked lever to mitigate HER aggravation. Ye *et al.*^97^ leveraged casein to regulate this coupling *via* abundant polar functionalities. Amino and carboxyl groups adsorb to form a hydrophilic barrier that physically limits water contact. Crucially, the emulsifying nature of casein enhances wettability to guide uniform Zn^2+^ transport. By alleviating the localized current density amplification associated with uneven growth, this mechanism kinetically suppresses gas evolution. This highlights that morphological uniformity fundamentally underpins the regulation of parasitic side reactions.

A related but mechanistically distinct mode is the post-formation management of interfacial by-products. In zinc–iodine batteries, the bioimmune-inspired Cy-H additive reconstructs the EDL, suppresses the HER, and cooperates with the local electric field and acidic environment to enable targeted elimination of alkaline by-products at the Zn anode interface (Fig. 4h).^98^ This process represents a scavenging-dominated mode, because its primary function is the chemical removal of accumulated interfacial species rather than the prevention of their access to active sites.

Overall, additive strategies in aqueous batteries have evolved from isolated parasitic-reaction suppression toward coordinated interfacial regulation. Rather than merely limiting water activity, current designs increasingly combine solvation control, hydrogen-bond reorganization, directed deposition, and by-product management to stabilize the interface and mitigate water-derived degradation.

#### 2.3.3 Corrosive-species management in high-voltage cathodes

By contrast, in high-voltage cathodes, electrolyte decomposition generates corrosive species that destabilize the cathode–electrolyte interface and accelerate structural degradation. Recent studies demonstrate that electrolyte additives can suppress these parasitic processes primarily through *in situ* scavenging and interphase reconstruction, thereby stabilizing interfacial chemistry under oxidative conditions.

To stabilize high-nickel interfaces, Forero-Saboya *et al.*^99^ introduced lithium tri-*tert*-butoxyaluminohydride (LTBA) as a single-molecule additive to stabilize high-nickel cathodes. The hydride moiety of LTBA consumes trace moisture and acidic species, suppressing HF accumulation and limiting transition-metal dissolution (Fig. 4i). Simultaneously, LTBA undergoes preferential oxidation to generate an Al-rich cathode-electrolyte interphase that passivates the surface under oxidative bias. This dual-function design effectively links corrosion suppression to interphase reinforcement. Building on this multifunctional strategy, Li *et al.*^100^ developed pentafluorophenyl diethoxy phosphate (FPOP) for NCM811-based cells. FPOP is preferentially oxidized ahead of carbonate solvents, producing a thin, compact CEI. Concurrently, it scavenges HF *via* coordination and irreversible fluorination pathways to suppress corrosive species accumulation (Fig. 4j). Furthermore, the phosphate motif imparts flame-retardant properties, positioning single-additive multifunctionality as a practical route to co-optimize CEI robustness and intrinsic safety.

Beyond single-molecule solutions, the strategic partitioning of chemical tasks between multiple additives or specialized salts offers a more granular approach to interfacial stabilization. Song *et al.*^101^ demonstrated this by stabilizing NCM622 at high cutoff voltages through a dual-additive strategy that decouples interphase construction from corrosive-species control. TPFPB preferentially participates in interfacial reactions to form a thin, uniform B- and F-containing CEI, which passivates the surface and suppresses sustained electrolyte oxidation. LiDFOB complements this protection by consuming trace protic impurities and strongly coordinating PF_5_, thereby attenuating LiPF_6_ derived corrosion chemistry and limiting HF-driven interfacial attack. This division of roles improves CEI persistence and cathode stability under oxidative bias.

To further refine high-voltage chemistry, Yang *et al.*^102^ stabilized high-voltage LiCoO_2_–Si/C cells using potassium tetracyanoborate (TCB), a hydrogen-free cyano-functional salt additive. The cyano-rich TCB anion preferentially adsorbs on Co sites, displacing solvent molecules to promote a cyano-derived CEI enriched with inorganic components like LiF. This reconstructed interphase effectively mitigates electrolyte oxidation and transition-metal leaching. Simultaneously, TCB neutralizes HF *via* ligand exchange and converts to BF_4_ (Fig. 4k). This study identifies ligand-engineered salts as a scalable handle to precisely tune high-voltage interfacial environments.

More importantly, strategies for regulating soluble and reactive intermediates are increasingly shifting from single-mechanism mitigation toward multifunctional and synergistic control. Rather than relying on isolated physical confinement or chemical adsorption, recent approaches integrate catalytic conversion, interphase engineering, and reactive-species management to interrupt parasitic pathways. Despite system-specific constraints, the central design objective is the selective and sustained regulation of reactive intermediates, which will be critical for translating laboratory strategies into practical, long-term stable battery systems.

## 3 Molecular additives in electrocatalytic systems

### 3.1 Activity tuning *via* interfacial microenvironment regulation

**Fig. 5:**
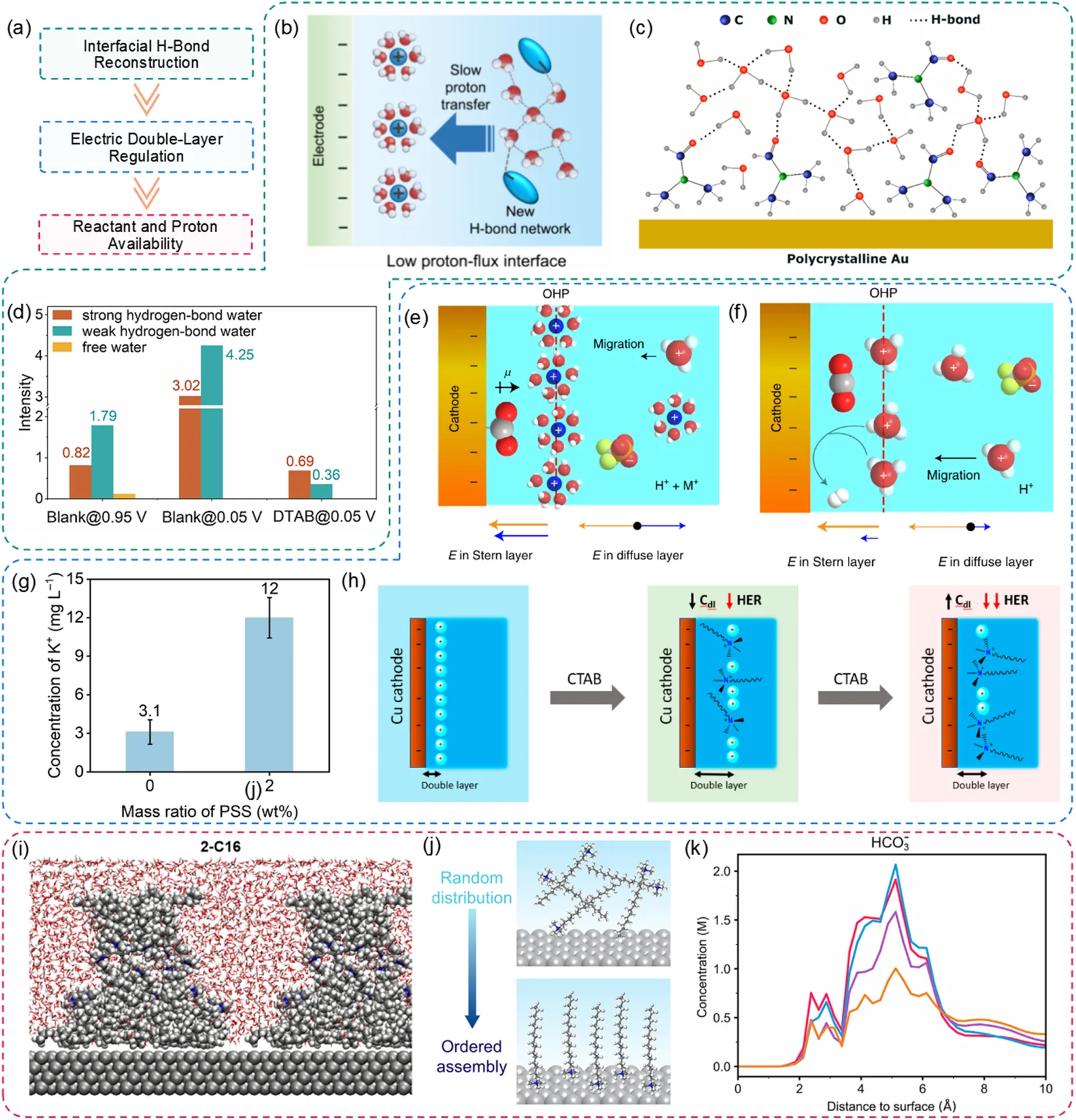
Interfacial microenvironment regulation by molecular additives in electrocatalysis. (a) Schematic overview of the three main modes of interfacial microenvironment regulation, including hydrogen-bond-network reconstruction, electric-double-layer regulation, and local reactant and proton availability control. (b) Schematic of the low-proton-flux interface induced by sulfonate-based additives in acidic CO_2_ electrolysis.^103^ Copyright 2024 American Chemical Society. (c) Interfacial model of the DMF-modified water structure near a polycrystalline Au surface.^104^ Copyright 2023 American Chemical Society. (d) Quantitative comparison of strong hydrogen-bond water, weak hydrogen-bond water, and free water in blank and DTAB-containing electrolytes.^105^ Copyright 2024 American Chemical Society. (e) Schematic of the double-layer structure and electric-field distribution in an acidic medium containing alkali cations.^106^ Copyright 2022 Springer Nature. (f) Schematic of the double-layer structure and electric-field distribution in an acidic medium without alkali cations.^106^ Copyright 2022 Springer Nature. (g) Interfacial K^+^ concentration in acidic electrolyte without and with a PSS additive.^107^ Copyright 2024 Wiley. (h) Proposed double-layer reorganization induced by CTAB accumulation at the Cu cathode interface.^108^ Copyright 2019 American Chemical Society. (i) Molecular dynamics snapshot of the near-surface environment created by 2-C16 on Ag.^109^ Copyright 2021 American Chemical Society. (j) Schematic of surfactant evolution from random distribution to ordered assembly under bias.^110^ Copyright 2022 American Chemical Society. (k) Concentration profile of HCO_3_^−^ as a function of distance to surface in the absence and presence of sorbitol.^111^ Copyright 2025 American Chemical Society.

Although electrolyte additives in electrocatalysis are often introduced through individual molecular examples, their influence on catalytic activity can usually be traced back to a limited set of interfacial microenvironment controls. In proton-involved electrocatalytic reactions, additives can reorganize hydrogen-bond connectivity, reshape the electric-double-layer structure, and alter the near-surface availability of reactants and proton-related species, thereby shifting the kinetic balance between target pathways and competing reactions. From this perspective, activity regulation can be understood through three coupled modes: hydrogen-bond-network reconstruction, electric-double-layer regulation, and local reactant and proton availability control (Fig. 5a).

#### 3.1.1 Interfacial hydrogen-bond-network reconstruction

Before direct adsorbate stabilization becomes dominant, electrolyte additives can already reshape electrocatalytic behavior by reorganizing the hydrogen-bond network of interfacial water. Since proton transfer at electrified interfaces depends strongly on water-mediated hydrogen-bond connectivity, perturbing this network provides a direct way to regulate proton delivery and shift the competition between parallel reaction pathways.

Sulfonate-based additives were shown by Ge *et al.*^103^ to reconstruct the interfacial hydrogen-bond environment in acidic CO_2_ electrolysis. SPS, STS, and SBS interact strongly with water, reduce proton flux near the electrode, and thereby suppress proton-dependent HER. A reorganized water network together with slowed proton transfer is directly conveyed by the selected low-proton-flux interfacial schematic, where additive-assisted hydrogen-bond rearrangement replaces the original dense proton-transport pathway (Fig. 5b).

A similar idea appears in the work of Mohandas *et al.*,^104^ but through a nonionic additive. By introducing DMF into aqueous KHCO_3_, they showed that the near-surface water structure on Au is altered and that strongly hydrogen-bonded DMF–water pairs become increasingly important at negative potentials, which correlates with suppressed HER and improved CO selectivity. The selected interfacial model captures this DMF-enriched region above polycrystalline Au and emphasizes that the key change occurs in the local water organization rather than in the catalyst itself (Fig. 5c).

Further support comes from surfactant additives under alkaline conditions. Fan *et al.*^105^ found that quaternary ammonium surfactants, especially DTAB, weaken the H_2_O-dominated interfacial hydrogen-bond network during the ORR, lower proton-transfer kinetics, and favor the two-electron pathway toward H_2_O_2_. This trend is reflected clearly by the selected quantitative comparison, where both strong and weak hydrogen-bond water decrease sharply after DTAB addition, particularly under bias, indicating substantial disruption of the interfacial water network (Fig. 5d). From the primitive-framework perspective, DTAB is categorized as additive-induced microenvironment remodeling with pathway competition: surfactant assembly disrupts interfacial hydrogen-bond connectivity and slows proton transfer, thereby favoring the two-electron ORR route over proton-transfer-dominated competing pathways.

Collectively, these results indicate that hydrogen-bond-network reconstruction serves as an effective first layer of additive regulation in electrocatalysis. Rather than primarily acting through direct surface binding, these additives first reshape the interfacial water structure and proton-transfer pathways, which then alter the kinetic preference of the overall reaction network.

#### 3.1.2 Electric-double-layer regulation

Besides reconstructing the hydrogen-bond network, electrolyte additives can also regulate electrocatalysis by reshaping the electric double layer. This level of control is especially important in proton-involved reactions, because the local field distribution and ion arrangement near the outer Helmholtz plane directly affect proton migration, reactant approach, and the competition between target reduction pathways and the HER.

At a conceptual level, Gu *et al.*^106^ clarified how added alkali cations redistribute the interfacial electric field in strongly acidic CO_2_ electrolysis. Their model shows that hydrated cations accumulated at the outer Helmholtz plane confine the field within the Stern layer and weaken it in the diffuse layer, thereby suppressing hydronium migration toward the cathode while still favoring CO_2_ reduction. The selected schematic captures this contrast clearly by comparing the cation-assisted interface with the cation-free case and showing how hydronium supply is restricted once the field is screened near the OHP (Fig. 5e and f).

Building on this idea, Wang *et al.*^107^ introduced polystyrene sulfonate as a polyanionic electrolyte additive for acidic CO_2_ electrolysis and showed that it enriches K^+^ at the electrode–electrolyte interface through electrostatic interactions. The selected bar chart gives a direct quantitative picture of this effect, with the interfacial K^+^ concentration increasing sharply after PSS is added, which supports the view that additive-assisted cation enrichment is a practical route to double-layer regulation rather than a purely conceptual model (Fig. 5g).

A related but chemically distinct strategy appears in the work of Banerjee *et al.*,^108^ who used CTAB as a cationic surfactant additive in CO_2_ reduction. Their impedance analysis suggests that CTAB accumulates in the double layer, perturbs the local ion distribution, and lowers the available proton sources for the HER. The selected mechanistic cartoon summarizes this progression well: initial CTAB addition disrupts the pre-existing double layer and decreases the HER, while further accumulation reorganizes the interfacial region more extensively and sustains stronger HER suppression (Fig. 5h).

Taken together, these studies show that electric-double-layer regulation is a second, distinct layer of microenvironment control. Rather than acting mainly through direct adsorption on catalytic sites, additives first reorganize near-surface cation populations, field distribution, and proton transport, which then shifts the kinetic balance of the reaction network.

#### 3.1.3 Local reactant and proton availability control

Electrolyte additives can further regulate electrocatalysis by changing which species are preferentially retained near the active surface. In aqueous CO_2_ reduction, the local balance among CO_2_, H_2_O, and proton-related species directly influences the competition between the CO_2_RR and HER.

Using long-chain quaternary ammonium additives on Ag, Buckley *et al.*^109^ showed that the most selective modifier, 2-C16, creates a more favorable near-surface reactant environment for CO formation. Their simulations indicate that high CO selectivity is associated with increased local CO_2_ availability while still preserving sufficient interfacial water for CO_2_ activation. The selected molecular snapshot reflects this additive-defined interfacial region, where the ordered organic layer redistributes the local reactant space above the Ag surface (Fig. 5i).

A related effect appears when surfactant additives reorganize under bias. Ge *et al.*^110^ found that quaternary ammonium surfactants gradually evolve from a random distribution to a nearly ordered assembly as the cathodic potential increases, and this structural transition promotes CO_2_ enrichment while suppressing unfavorable interfacial water organization. The selected schematic directly captures this evolution from disordered adsorption to ordered assembly at the electrified interface (Fig. 5j).

An alternative route is provided by a nonionic additive. In a practical GDE flow-cell system, Sihag *et al.*^111^ demonstrated that sorbitol improves CO selectivity and suppresses the HER, while molecular dynamics analysis revealed a marked decrease in bicarbonate concentration near the electrode interface. The selected concentration profile shows this change clearly and supports the view that lowering the local HCO_3_^−^ population can help weaken proton-related side reactivity at the surface (Fig. 5k).

Altogether, these examples show that local reactant and proton availability is not fixed by the bulk electrolyte alone. By enriching CO_2_ and reducing the interfacial presence of species that favor proton-related side reactions, additives can shift the near-surface environment toward the target electrocatalytic pathway.

### 3.2 Pathway steering *via* adsorbate and surface-state regulation

**Fig. 6:**
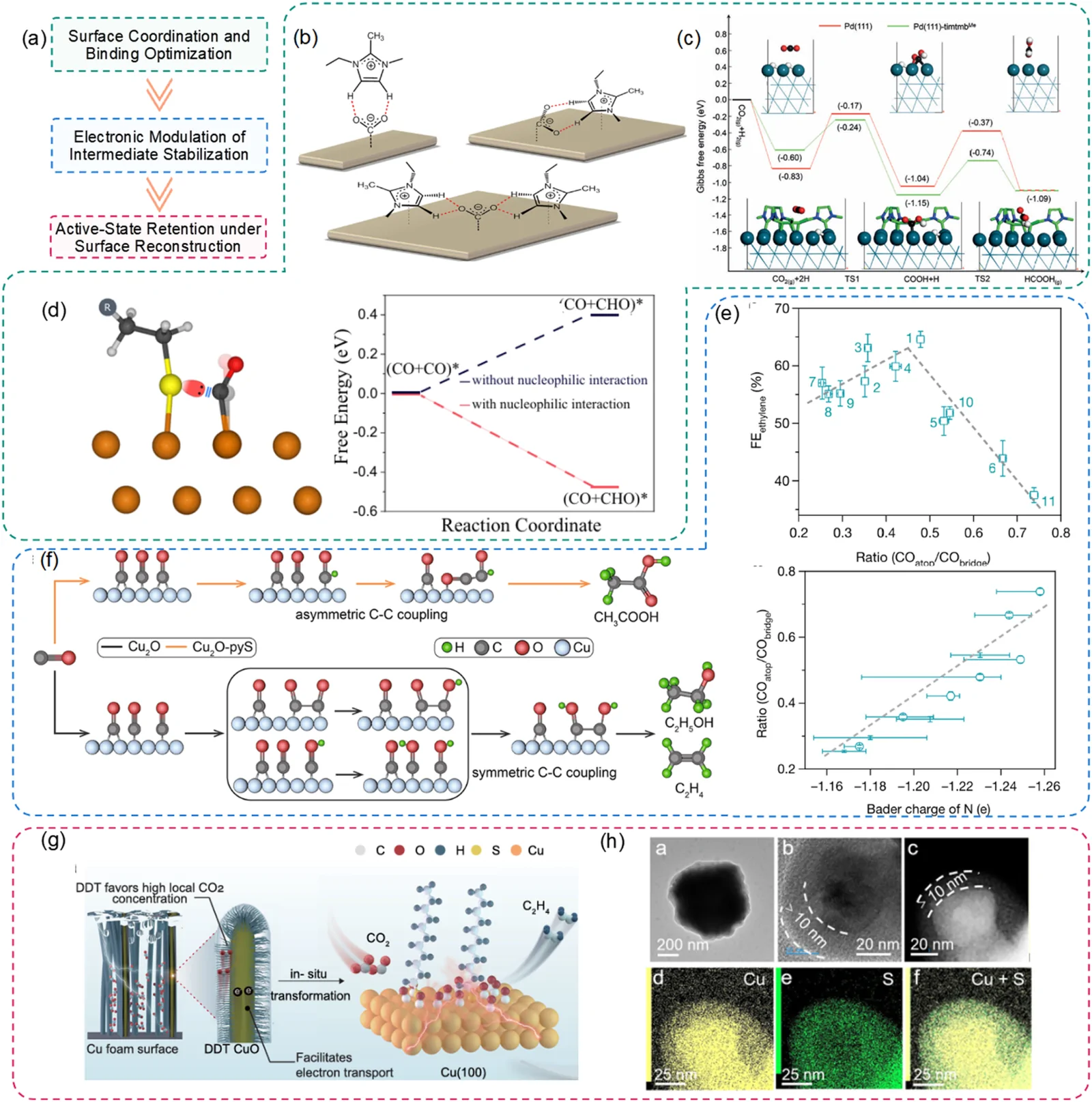
Adsorbate stabilization and surface-state regulation *via* molecular modifiers. (a) Schematic overview illustrating the hierarchical regulation strategies: surface coordination, electronic modulation, and dynamic active-state retention. (b) Illustration of the hydrogen-bonding network between an imidazolium cation and the electrogenerated 
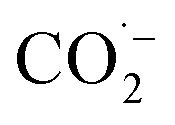
 radical anion on a Ag electrode.^113^ Copyright 2016 American Chemical Society. (c) Schematic of the local coordination geometry showing a chelating NHC ligand on Pd that lowers the energy barrier for *COOH formation.^114^ Copyright 2018 Wiley-VCH. (d) Mechanism scheme showing the orbital interaction and hybridization shift of *CO toward sp^2^ induced by the nucleophilic lone-pair electrons of thiol ligands on Cu.^115^ Copyright 2024 Springer Nature. (e) Correlation trend between the ethylene faradaic efficiency and calculated Bader charge, illustrating the p-doping effect induced by *N*-arylpyridinium additives on Cu.^117^ Copyright 2019 Springer Nature. (f) Schematic illustrating the asymmetric C–C coupling pathway toward acetate promoted by surface-modified pyridine derivatives.^119^ Copyright 2024 Springer Nature. (g) Schematic showing DDT molecules acting as a surface stabilizer for the highly active Cu(100) facet to enhance *CO coverage and mass transfer.^122^ Copyright 2024 Springer Nature. (h) Cross-sectional illustration of a >10 nm thick metal–organic interphase (MOI) formed on Cu, sustainably maintaining the specific coordination state for complex pathway control.^123^ Copyright 2025 Springer Nature.

While regulation of the outer interfacial water network and electric double layer establishes a favorable macroscopic microenvironment, atomistic control over reaction pathways requires the direct stabilization of key adsorbates and catalytically favorable surface states. In contrast to simple physical blockers, surface-anchored molecular modifiers function as active participants at the inner catalytic interface. From this perspective, the following discussion proceeds through three interconnected levels of regulation: surface coordination and binding optimization, electronic modulation of intermediate stabilization, and active-state retention under surface reconstruction (Fig. 6a). Accordingly, the examples in this section are distinguished by their dominant regulatory target: spectator-species surface coverage, pathway-relevant intermediate stabilization, or retention of catalytically favorable surface states.

#### 3.2.1 Surface coordination and binding optimization

Surface molecular modification is not merely steric blocking; rather, it reshapes the local geometric environment and optimizes adsorption configurations through specific interactions with catalytic sites or key intermediates. At the electrocatalytic interface, this regulation can appear either as surface-coverage control, such as excluding competitive spectator species, or as direct stabilization of reaction intermediates through promoter–surface–adsorbate interactions. For example, in alkaline hydrogen evolution and oxidation reactions (HER/HOR), a caffeine molecular layer adsorbed on the platinum surface can effectively weaken the binding energy of hydroxyl (OH*), significantly reducing the oxophilicity of the surface, thereby optimizing the adsorption–desorption kinetics of hydrogen species.^112^ Moving beyond the steric exclusion of spectator species, the molecular modification layer can more directly participate in the stabilization of target intermediates. In the carbon dioxide reduction system on a silver electrode, an imidazolium cation with a specific configuration utilizes the protons at the C_4_ and C_5_ positions on its imidazole ring to directly anchor and stabilize the electrogenerated 
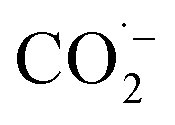
 radical anion *via* a non-covalent hydrogen-bonding network, thereby establishing a direct synergistic stabilization motif involving the promoter, surface, and reactant (Fig. 6b).^113^

Furthermore, through the strategy of “surface organometallic chemistry”, strong covalent ligands can fundamentally alter the local coordination geometry of metal atoms. The introduction of multidentate N-heterocyclic carbene (NHC) ligands onto the palladium surface leverages the strong coordination interaction formed by the chelate effect to not only improve interfacial stability but also significantly lower the energy barrier for the formation of the *COOH intermediate, thereby guiding the CO_2_ reduction pathway toward formate with high selectivity (Fig. 6c).^114^ This molecular-level interfacial coordination can even extend to altering the electron orbital hybridization state of intermediates. On the surface of copper nanocatalysts, thiol ligands act not only as an anchoring layer, but the lone-pair electrons of their sulfur atoms can undergo nucleophilic interactions with the empty orbitals of key intermediates such as adsorbed carbon monoxide (*CO) (Fig. 6d).^115^ This cross-interface orbital overlap leads to the polarization of the C–O and Cu–C bonds, promoting a shift in the hybridization state of *CO toward sp^2^, which substantially lowers the reaction barrier of the rate-determining step (CO to CHO conversion). Concurrently, this coordination environment specifically stabilizes the (HOOC–CH_2_) intermediate, the key precursor to acetate formation, rather than the (HOHC–CH_2_O) intermediate that leads to ethylene, achieving highly efficient directed conversion to C_2_ oxygenates. Collectively, these studies demonstrate that optimizing the local binding environment through surface coordination serves as the primary physicochemical foundation for achieving adsorbate stabilization and precise regulation of reaction pathways.

#### 3.2.2 Electronic modulation of intermediate stabilization

Building upon the optimization of the local coordination environment, molecular modification layers further systematically tune the binding energies of reaction intermediates by inducing interfacial charge redistribution or perturbing the metal's energy band structure, thereby precisely steering reaction pathways. In the HER, Zhao *et al.* proposed a universal molecular design strategy by quantifying the correlation between the binding energy of the organic layer and the Pt surface d-band center.^116^ This enabled continuous modulation of the hydrogen adsorption energy (Δ*G*_H*_), successfully surpassing the conventional activity limits of traditional metal catalysts.

Such electronic perturbations are likewise the core mechanism for controlling selectivity in the CO_2_RR. Li *et al.* found that arylpyridinium molecules can significantly alter the electron density of the copper surface through a “p-doping” effect.^117^ This electronic modulation effectively stabilizes atop-bound CO, thereby markedly enhancing ethylene production efficiency, as evidenced by the strong correlation between calculated Bader charges and adsorption energies (Fig. 6e). Additionally, Wu *et al.* utilized nitrogen-containing heterocycles with strong inductive effects to induce Cu^*δ*+^ active sites on the Cu surface.^118^*In situ* spectroscopic and Bader charge analyses confirmed that this electronic perturbation not only strengthened the binding of atop CO but also significantly lowered the kinetic energy barrier for C–C coupling. This intermediate-specific electronic stabilization ultimately enables precise guidance of complex product pathways, as demonstrated by Ding *et al.*, who employed pyridine derivatives to induce asymmetric adsorption states of intermediates, specifically promoting the coupling pathway toward acetate (Fig. 6f).^119^

Overall, electronic-structure modulation provides a second route to adsorbate regulation: instead of only changing surface coverage, molecular modifiers tune charge distribution, d-band position, and intermediate binding strength, thereby stabilizing pathway-relevant adsorbates and steering selectivity.

#### 3.2.3 Active-state retention under surface reconstruction

Under operating potentials, catalyst surfaces remain in a dynamic state of evolution, where atomic diffusion, surface reconstruction, and oxidation-state fluctuations continuously reshape the distribution of active sites. Rather than merely stabilizing adsorbates locally, molecular modifiers can direct this evolution by transforming disordered reconstruction into controlled surface-state retention. The following examples highlight molecular regulation of *in situ* reconstruction, facet stabilization, and preservation of surface states that sustain selective reaction pathways. This category therefore differs from intermediate stabilization because the primary target is not an individual adsorbed species, but the persistence of an active surface configuration under reaction conditions. Thevenon *et al.* proposed using an organic salt additive (*e.g.*, *N*,*N*′-ethylene-phenanthrolinium dibromide) to guide the *in situ* nanostructuring of polycrystalline copper surfaces during the reaction. Through a surface corrosion and redeposition mechanism, highly stable nanocube structures were formed, which not only significantly enhanced ethylene selectivity but also maintained high morphological and catalytic stability over 40 hours of continuous electrolysis.^120^ This guiding effect further enables precise control over the direction of reconstruction. Han *et al.* successfully suppressed the random coarsening of the copper surface through the strong chelating effect of an electrolyte additive (EDTMPA) with copper ions, inducing its reconstruction toward active sites favorable for methane production, thereby achieving the precise redirection and retention of the active state.^121^ Beyond morphological regulation, the locking of metastable facets by the molecular layer is also crucial for maintaining high-activity states. Yao *et al.* modified CuO-derived copper catalysts with dodecanethiol (DDT) and found that thiol molecules not only facilitated CO_2_ transfer but also specifically stabilized the highly active Cu(100) facet through strong surface anchoring (Fig. 6g). By increasing *CO coverage and lowering the energy barrier for asymmetric C–C coupling, this strategy dramatically boosted the ethylene selectivity to 79.5%.^122^ The ultimate evolutionary form of surface regulation is the construction of an interphase with a substantial thickness on the catalyst surface. Through high-throughput experimental screening, Shen *et al.* discovered that in highly ethanol-selective systems, a metal–organic interphase (MOI) over 10 nm thick formed on the copper surface (Fig. 6h). Unlike traditional single-molecule adsorption layers, this MOI can sustainably maintain specific coordination states of copper and regulate the distribution of interfacial water molecules, thereby achieving durable and stable control over complex pathways (*e.g.*, CO_2_-to-ethanol) on a macroscopic time scale.^123^

Taken as a whole, these studies show that molecular modifiers can help preserve catalytically favorable surface states during reconstruction rather than merely suppressing structural change. In this way, surface-state retention becomes an extension of adsorbate stabilization, because maintaining the right local geometry and coordination environment is often essential for sustaining selective reaction pathways under operating conditions.

### 3.3 Selectivity enhancement *via* competing-reaction suppression

**Fig. 7:**
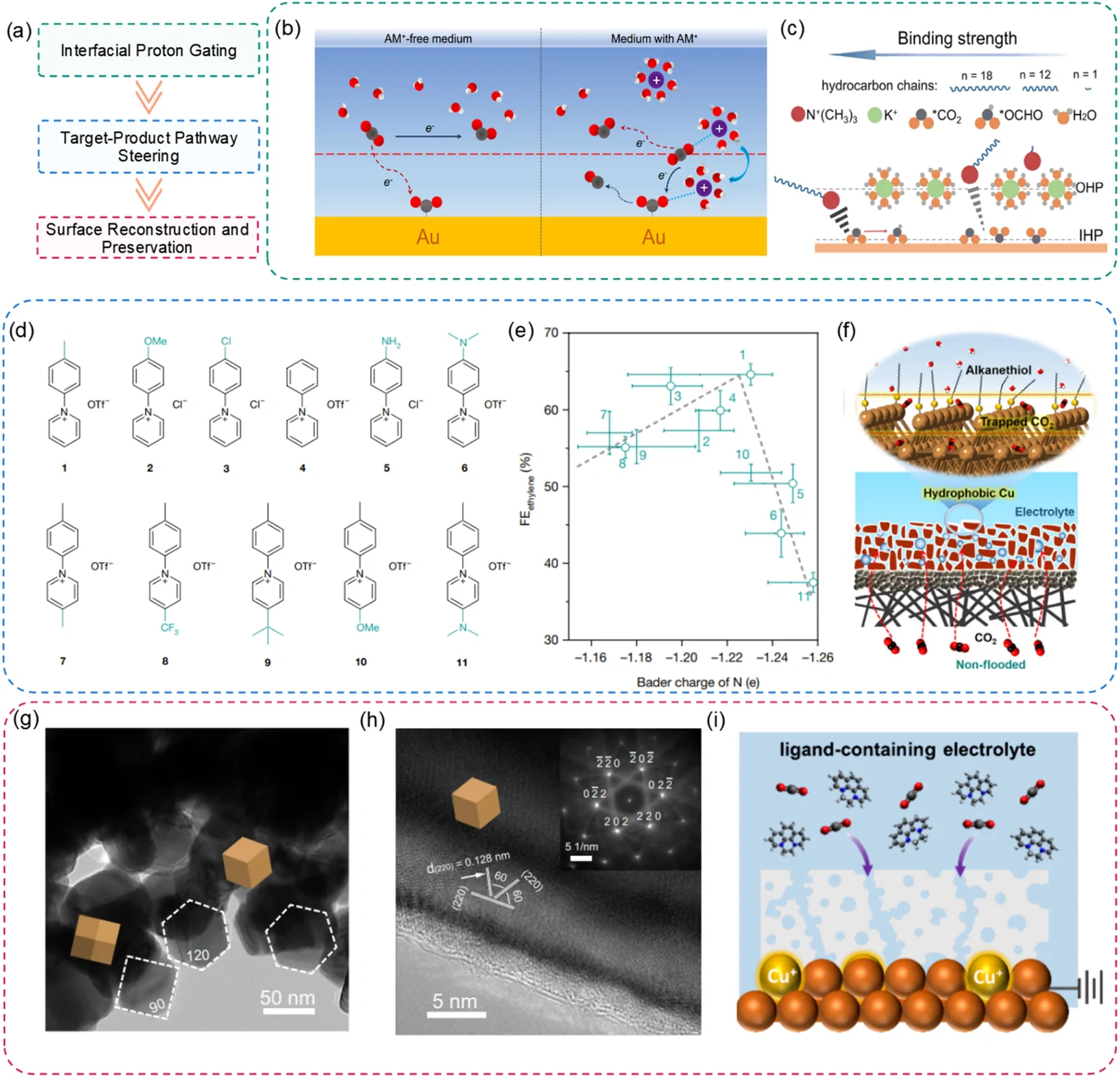
Dynamic interfacial gatekeepers for enhancing selectivity and catalyst durability. (a) Schematic overview of the hierarchical logic flow, from interfacial proton gating to target-product pathway steering and surface reconstruction control for catalyst preservation. (b) Schematic illustration of the CO_2_RR at Au–water interfaces showing the transition from OS-ET to IS-ET mediated by cations.^124^ Copyright 2023 Springer Nature. (c) Mechanism scheme illustrating the steric effect of C_*n*_TA^+^ cations with varying backbone lengths on tuning the binding strength of the intermediate.^125^ Copyright 2025 Wiley. (d) Molecular structures of *N*-arylpyridinium additives.^117^ Copyright 2019 Springer Nature. (e) Trend for ethylene FE and calculated Bader charge for the nitrogen atom of the *N*-aryl-substituted tetrahydro-bipyridines.^117^ Copyright 2019 Springer Nature. (f) Illustration of microenvironments and the local reaction interface of a modified GDE with 1-octadecanethiol functionalized Cu.^126^ Copyright 2022 Springer Nature. (g) TEM image of an electrodeposited Cu TEM grid after electrocatalysis in the EDTMPA-added electrolyte.^121^ Copyright 2022 Springer Nature. (h) HRTEM image showing the corresponding SAED pattern and reconstructed facets.^121^ Copyright 2022 Springer Nature. (i) Schematic illustration of the layer-modified Cu electrode surface in the modifier-containing electrolyte.^127^ Copyright 2024 American Chemical Society.

Beyond amplifying intrinsic activity, the practical value of electrocatalysts depends on suppressing competing reactions and degradation pathways that weaken both selectivity and lifetime. Molecular additives can serve as dynamic interfacial gatekeepers by regulating proton accessibility, adsorbate conversion, and surface coordination through steric blocking, hydrophobicity, and selective binding. Accordingly, this section discusses competing-reaction suppression at three levels: limiting proton-transfer pathways that lead to the HER, redirecting carbon-containing intermediates away from non-target hydrogenation routes, and preserving active surface states against reconstruction-induced degradation (Fig. 7a).

#### 3.3.1 Interfacial gating of competitive proton transfer

In CO_2_ and related electrocatalytic reactions, competition between proton transfer and substrate activation is strongly governed by the structure of the electrical double layer. As elucidated by Qin and co-workers,^124^ alkali cations are not passive spectators but active drivers that dictate the reaction pathway (Fig. 7b). By shifting CO_2_ activation from a prohibitive outer-sphere mechanism to a feasible inner-sphere electron transfer, these cations significantly lower the activation barrier for CO_2_ adsorption relative to proton transfer, thereby establishing a thermodynamic preference for the target reaction at its kinetic origin.

While inorganic cations operate primarily *via* field effects, bulky organic additives introduce precise steric regulation. Bulky quaternary ammonium cations can restructure the Helmholtz plane and impose steric regulation, as demonstrated by Han *et al.*^125^ (Fig. 7c). These bulky molecules create a physical barrier that restricts proton access for the HER while selectively stabilizing formate intermediates *via* electrostatic interactions. This “steric-dominated” mechanism effectively filters out competitive protons, steering selectivity toward carbonaceous products through simple yet effective spatial exclusion.

Taken together, these studies show that competing proton transfer can be suppressed at its interfacial origin. Whether through cation-mediated field effects or steric gating by bulky organic cations, additives reduce proton access and establish an early kinetic preference for carbon-centered pathways.

#### 3.3.2 Pathway steering toward target carbon products

Beyond HER suppression, additives can also redirect carbon-containing intermediates toward specific products by limiting non-target hydrogenation and other product-diverting pathways. Steering more complex C–C coupling processes therefore requires stabilizing selected intermediates while suppressing competing conversion routes that divert them from the desired carbon products. Li *et al.*^117^ addressed this by functionalizing the electrode interface with a library of *N*-arylpyridinium additives (Fig. 7d). These molecules go beyond simple site-blocking; they actively stabilize multipoint-bound intermediates essential for dimerization. As evidenced by the correlation between the nitrogen Bader charge and ethylene selectivity (Fig. 7e), tuning the electronic properties of the additives effectively lowers the energy landscape for C–C coupling, ensuring that electron flux is channeled toward high-value ethylene rather than thermodynamically favorable hydrogen evolution.

Complementing these electronic and steric modulations, constructing a physical barrier offers a direct way to manage reactant flux. Xue *et al.*^126^ constructed a “molecular shield” by assembling long-chain thiols on copper surfaces to manage interfacial water availability (Fig. 7f). This superhydrophobic layer drastically reduces water concentration at the active sites while maintaining high CO_2_ permeability. Consequently, hydrogen evolution is stifled by reactant limitation, enabling robust catalytic performance and high faradaic efficiency even at elevated current densities where parasitic currents typically dominate.

These examples show that selectivity enhancement is not achieved solely by blocking the HER, but by suppressing non-target hydrogenation and product-diverting pathways that redirect intermediates away from the desired carbon products. By stabilizing key intermediates or reshaping local reactant flux, additives can channel the reaction network toward higher-value products under demanding operating conditions.

#### 3.3.3 Surface reconstruction control and active-site preservation

In addition to molecular side reactions, competing processes in electrocatalysis can also originate from structural evolution of the catalyst surface. Uncontrolled reconstruction, loss of favorable facets, and deterioration of active coordination states may redirect reaction pathways or accelerate deactivation under operating potentials. Once selectivity is established, durability depends on whether the catalyst can retain its active surface under reaction conditions. Beyond competitive reactions, parasitic degradation often manifests as uncontrollable surface reconstruction. Han *et al.*^121^ demonstrated that this thermodynamic tendency can be inverted into a stabilization strategy (Fig. 7g and h). By introducing phosphonic acid additives, the reconstruction of polycrystalline copper is guided into specific, stable facets favorable for methane production. This approach transforms a potential degradation mechanism into a “constructive reconstruction,” dynamically locking the active surface morphology against deactivation.

Ultimately, the chemical fidelity of the active site determines longevity. Peng *et al.*^127^ established that *in situ* formed organic thin films function as essential protective barriers against chemical degradation (Fig. 7i). By physically isolating the copper surface and providing coordinating stabilization, these films enable the retention of crucial Cu(i) species that would otherwise be reduced to inactive metallic copper under harsh reducing potentials. This finding underscores the dual role of additives: simultaneously tuning selectivity and inhibiting corrosion to preserve the catalyst's functional lifetime.

Overall, competing-reaction suppression in electrocatalysis has evolved from simple HER inhibition toward a broader strategy that integrates proton gating, product-pathway steering, and active-site preservation. This progression highlights that durable selectivity depends not only on suppressing unwanted reactions, but also on maintaining the interfacial states that sustain the desired catalytic route.

## 4 Machine learning for molecular additive discovery and design

Before turning to data-driven discovery strategies, it is necessary to place the representative additive examples discussed above into a comparative framework. Molecular additives and electrolyte modifiers in batteries and electrocatalysis are evaluated using distinct performance descriptors, including cycling stability, coulombic efficiency, faradaic efficiency, product selectivity, and operational durability. Therefore, Table 1 does not aim to establish a direct numerical ranking across different electrochemical systems. Instead, it summarizes selected representative examples according to their application scenarios, regulated interfacial targets, dominant molecular functions, and reported outcomes, thereby providing a mechanistic bridge between case-based additive studies and the data-driven design principles discussed below.

**Table 1:** Representative molecular additives and modifiers for interfacial regulation in batteries and electrocatalysis

Additive	Application	Regulated target	Dominant function	Representative outcome	Ref.
NDPA	Aqueous Zn metal battery	Zn^2+^ solvation shell; desolvation barrier	Coordination remodeling	Lowered desolvation penalty and facilitated reversible Zn plating/stripping	66
BMIm^+^	Aqueous Zn metal battery	High-reactivity Zn facets; deposition orientation	Facet-selective adsorption	Suppressed dendrite growth and promoted Zn (002)-oriented deposition	71
DMIO	Lithium metal battery	SEI/CEI chemistry; acidic impurities	Bipolar interphase engineering	Concurrent SEI/CEI construction under high-voltage operation	83
TCPy	Lithium–sulfur battery	Soluble polysulfides; shuttle pathway	Covalent anchoring and interfacial blocking	Insoluble cross-linked interphase for polysulfide confinement	90
l-Leucine	Lithium–sulfur battery	Li_2_S_*x*_ adsorption; Li_2_S formation	Dynamic anchoring and catalytic conversion	Coupled polysulfide immobilization with accelerated sulfur redox kinetics	91
Erythritol	Aqueous Zn battery	Zn^2+^ solvation; hydrogen-bond network; Zn surface	Water-activity regulation	Suppressed water-induced parasitic reactions and promoted Zn^2+^ transport	92
Cy-H	Zinc–iodine battery	Alkaline by-product accumulation; parasitic HER	By-product scavenging	Targeted elimination of accumulated alkaline by-products at the Zn interface	98
Imidazolium cation	CO_2_RR on Ag	Activated 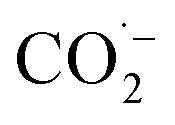 intermediate	Intermediate stabilization	Stabilized early CO_2_ activation through local ionic interactions	113
NHC ligand	CO_2_RR on Pd	*COOH formation pathway	Surface coordination	Stabilized pathway-relevant intermediates for formate formation	114
Thiol ligand	CO_2_RR on Cu	*CO and C_2_ oxygenate-related intermediates	Ligand-induced adsorbate modulation	Directed C_2_ oxygenate formation through ligand–adsorbate interactions	115
*N*-Arylpyridinium additive	CO_2_RR on Cu	Cu electron density; atop-bound CO	Electronic modulation	Enhanced ethylene formation through CO-intermediate stabilization	117
DDT/metal–organic interphase	CO_2_RR on Cu	Cu(100) facet; Cu coordination state; interfacial water	Surface-state retention	Preserved active Cu surface states for C_2+_ product formation	122 and 123

### 4.1 Data mining and predictive modeling

**Fig. 8:**
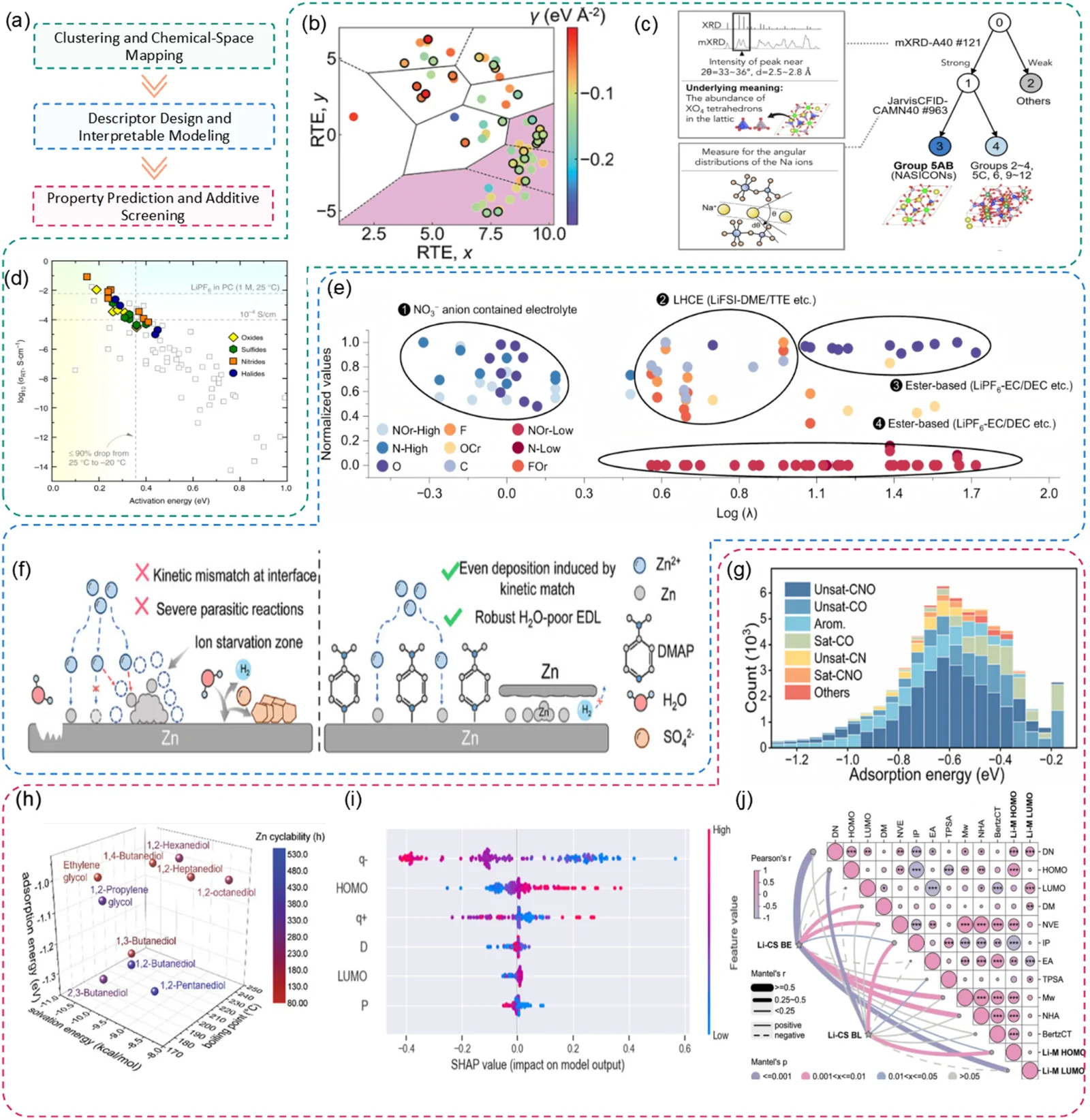
Data mining and predictive modeling for molecular additive discovery. (a) Schematic overview of the workflow from clustering and mapping to descriptor modeling and additive screening. (b) Random-trees embedding (RTE) map with Voronoi partitioning visualizing the additive design space based on surface free energy.^130^ Copyright 2024 Wiley. (c) *Post-hoc* decision-tree rules classifying NASICON-like families in Na conductors using interpretable descriptors.^131^ Copyright 2024 Springer Nature. (d) Ionic conductivity *versus* activation energy plot highlighting the high-*σ*/low-*E*_a_ region for Li conductors.^132^ Copyright 2019 Springer Nature. (e) Clustering of normalized SEI descriptors as a function of log(*λ*), identifying the low-*λ* region associated with favorable SEI chemistry and spherical Li deposition.^133^ Copyright 2025 Springer Nature. (f) Schematic illustrating the “global kinetics balance” mechanism, in which DMAP adsorption mitigates kinetic mismatch and promotes uniform Zn deposition.^134^ Copyright 2025 Wiley. (g) Distribution of GNN-predicted adsorption energies for >67k molecules on Zn (002) supporting preferential passivation.^135^ Copyright 2025 Wiley. (h) Low-dosage performance of alkanediols projected in a 3D descriptor space (adsorption, solvation, and association).^136^ Copyright 2024 Wiley. (i) SHAP analysis of a donor-number (DN) model identifying *q*^−^ and the HOMO as dominant factors.^137^ Copyright 2025 American Chemical Society. (j) Correlation mapping linking DN with Li^+^-component binding energy and frontier orbital levels.^138^ Copyright 2025 American Chemical Society.

The elusive mapping between the molecular structure and interfacial mechanisms has long hindered rational additive selection. Machine learning (ML) is increasingly being used to address this challenge by establishing a “predictive engine” that shifts the discovery paradigm from empirical trial-and-error toward quantitative design. This workflow moves from clustering and chemical-space mapping to descriptor design and interpretable modeling, and finally to property prediction and additive screening (Fig. 8a). By mapping molecular diversity onto complex interfacial processes, this ML-driven approach establishes a rigorous, predictive landscape for mechanism-guided additive discovery.

The reliability of such workflows depends not only on model architecture, but also on the provenance and comparability of the underlying data. Additive-related datasets are usually assembled from heterogeneous sources, including literature-mined formulation records, DFT-derived molecular or interfacial descriptors, experimental performance measurements, and curated database entries. Literature-mined records can broaden chemical coverage, as demonstrated by automatically generated battery-materials databases extracted from the literature, but they also require careful curation because reporting standards and metadata completeness vary across studies.^128^ Computed descriptors provide controlled electronic, adsorption, or solvation quantities, but their transferability depends on the interfacial model, solvation treatment, potential reference, and calculation protocol. Experimental records are most directly linked to practical cell or catalytic performance, yet they remain sensitive to the electrolyte composition, additive concentration, electrode preparation, testing protocol, and normalization method. More broadly, the battery data genome initiative highlights that standardized data hubs, provenance tracking, and interoperable metadata are essential for making heterogeneous battery data reusable in machine-learning workflows.^129^

Accordingly, heterogeneous additive data should be integrated through source-aware normalization and validation, rather than pooled as interchangeable training points. For machine-learning-ready additive datasets, experimental records should retain additive identity, concentration, electrolyte composition, electrode or catalyst preparation, cell or reactor configuration, potential or voltage scale, temperature, testing protocol, normalization method, and reproducibility information. Theoretical records should report molecular descriptors, adsorption or solvation energetics, interfacial model details, solvation treatment, potential reference, computational settings, and links to experimental validation whenever available.

#### 4.1.1 Clustering and chemical-space mapping

Clustering provides a practical starting point for organizing chemically diverse additives into reaction-relevant regions of interest. Unsupervised learning acts as a vital tool to organize complex additive chemistries. Li *et al.*^130^ applied this strategy to aqueous zinc-ion batteries by projecting surface free energy and polarity descriptors into a visual landscape. This design space, mapped *via* random trees embedding and Voronoi partitioning (Fig. 8b), identifies a stability-favored domain that effectively separates potent additives from inactive ones. This analysis successfully identified polyalcohols like 1,2,3-butanetriol as potent dendrite suppressors, demonstrating that structural clustering can autonomously predict interfacial stability without exhaustive testing.

A similar idea was further extended by Park *et al.*,^131^ who combined density-based spatial clustering with *post-hoc* decision-tree analysis to accelerate the discovery of sodium-ion conductors. This approach distinguished NASICON-like families with a small set of interpretable descriptors and achieved high classification purity (Fig. 8c). Together, these studies show that low-data clustering and simple rule extraction can efficiently narrow the search space and provide a useful methodological reference for molecular additive discovery in liquid systems.

A similar strategy has also been used to identify high-performance transport windows in solid-state systems. For example, Zhang *et al.*^132^ combined XRD fingerprints with clustering to identify fast lithium conductors and located a target quadrant characterized by high ionic conductivity and low activation energy (*E*_a_) (Fig. 8d).

Taken together, these studies show that clustering can efficiently narrow chemical space before detailed modeling begins. By identifying stable regions, recurring structure families, and target performance windows, it provides a practical starting point for data-driven additive discovery.

#### 4.1.2 Descriptor design and interpretable modeling

After chemical-space mapping, descriptor construction becomes the key step for linking the molecular structure to interfacial function. In this stage, machine learning is most useful when it extracts compact and interpretable variables that retain mechanistic meaning, rather than only classifying candidate molecules based on similarity. To clarify the relationship between model selection and descriptor construction, Table 2 summarizes representative machine-learning model families and descriptor types used for additive discovery in batteries and electrocatalysis.

**Table 2:** Representative machine-learning model families and descriptor types for additive discovery in batteries and electrocatalysis

Application	Model family	Descriptor type
Batteries	Clustering and dimensionality reduction	Molecular fingerprints, functional groups, and physicochemical descriptors
Batteries	Tree-based and interpretable models	HOMO/LUMO, dipole moment, donor number, and solvation energy
Batteries	Graph-based models	Molecular graphs, atom/bond features, charge descriptors, and learned molecular embeddings
Electrocatalysis	Regression and surrogate models	Adsorption energy, reaction free energy, d-band center, charge and coordination descriptors
Electrocatalysis	Graph-based and atomistic learning models	Atomic structures, adsorbate configurations, local coordination environments, and learned embeddings
Batteries and electrocatalysis	Active-learning and generative workflows	Latent molecular embeddings, target-property constraints, and uncertainty estimates

These model families differ in their balance between interpretability, data demand, and extrapolation capability. Clustering and dimensionality-reduction methods are useful for organizing chemical space, whereas tree-based models are more suitable for ranking chemically interpretable descriptors from limited datasets. Graph-based models offer a flexible route to learn molecular or interfacial representations directly from the structure, but their reliability depends strongly on dataset diversity and validation. For candidate generation, active-learning and generative workflows should be constrained by practical additive requirements, including electrochemical stability, solubility, ion transport, interfacial affinity, safety, and synthetic accessibility. The descriptor-based screening examples discussed below further illustrate how these model families can be connected to experimentally testable additive design rules.

Beyond molecular-level screening, descriptor construction can also be extended to additive-derived interphases, where the goal is to connect interphase chemistry with electrochemical morphology. To connect SEI chemistry with lithium growth behavior, Lu *et al.*^133^ introduced the concept of SEI omics and proposed the morphological descriptor *λ* to quantify crystal growth dimensionality. Their analysis identified an optimal low-*λ* region enriched in nitrogen and depleted in oxygen, which correlates with a high-width growth mode and the formation of spherical lithium deposits (Fig. 8e). This work shows how data-driven descriptors can move beyond simple composition tracking and instead capture structure–morphology relationships that are directly relevant to additive selection.

A more compact example was provided by Han *et al.*,^134^ who identified the highest occupied molecular orbital energy as a decisive descriptor for additive selection. On this basis, 4-dimethylaminopyridine was recognized as an effective dendrite suppressor. Its adsorption creates a water-deficient double layer, aligns charge-transfer rates with mass-transport supply, and relieves interfacial kinetic mismatch, thereby enabling more uniform zinc deposition (Fig. 8f). Here, the value of the descriptor lies not only in its screening power, but also in its direct connection to interfacial transport behavior.

Together, these studies show that descriptor design is most useful when it remains closely linked to the mechanism. By connecting molecular features with interfacial behavior, such descriptors move beyond simple classification and become practical criteria for additive discovery.

#### 4.1.3 Property prediction and additive screening

With descriptors in hand, the workflow can move from interpretation to screening. This transition becomes clear when descriptor-based reasoning is combined with quantitative prediction. Xu *et al.*^135^ coupled DFT calculations with graph neural network models to predict adsorption energies on Zn (002), reduction potentials, and aqueous stability across a large additive space. By comparing predicted adsorption energies with redox potentials relative to the water window, they defined a safe region containing candidates with both favorable adsorption characteristics and sufficient electrochemical stability (Fig. 8g). Shifting focus to low dosage efficacy, Shang *et al.*^136^ utilized a data-informed strategy to identify potent additives such as trifluoroacetamide. By projecting candidates into a 3D descriptor space defined by adsorption strength, solvation interaction, and intermolecular association, they identified a specific domain correlated with extended cycling lifetime (Fig. 8h). This mapping pinpoints the optimal feature combinations necessary for interfacial stability. When integrated with random forest analysis to quantify feature importance, these explicit physical descriptors provide a robust framework for discovering cost-effective formulations that maintain high performance even at minimal concentrations.

A more descriptor-focused route was demonstrated by Luo *et al.*,^137^ who established the Gutmann donor number (DN) as a unified solvation descriptor for electrolyte additives. By training ensemble machine learning models on molecular fingerprints, they enabled rapid DN prediction to identify candidates capable of modulating Zn^2+^ solvation and enhancing anode stability. Feature importance analysis identified the most negative atomic charge as the dominant predictor, followed by secondary contributions from the highest occupied molecular orbital (*E*_HOMO_) and the most positive atomic charge (Fig. 8i). This work demonstrates that a single, mechanistically interpretable descriptor facilitates large-scale screening that is both efficient and transparent.

To achieve rigorous transparency, Gao *et al.*^138^ integrated Gutmann donor number (DN) screening with SHAP analysis to provide mechanistic transparency for additive selection. Intrinsic donor ability correlates strongly with frontier orbital energies and the binding metrics of Li-molecule complexes (Fig. 8j), confirming that DN values dictate the stabilization of solvation structures. By elucidating how electronic parameters govern solvation shell reconstruction and surface passivation, this study establishes clear, mechanism-guided criteria for identifying additives that effectively stabilize Zn anodes.

Overall, this stage moves additive discovery from descriptor interpretation to practical candidate selection. By combining quantitative prediction with physically meaningful screening criteria, machine learning can narrow large molecular spaces into smaller sets of promising additives for experimental validation.

### 4.2 Screening and closed-loop optimization

**Fig. 9:**
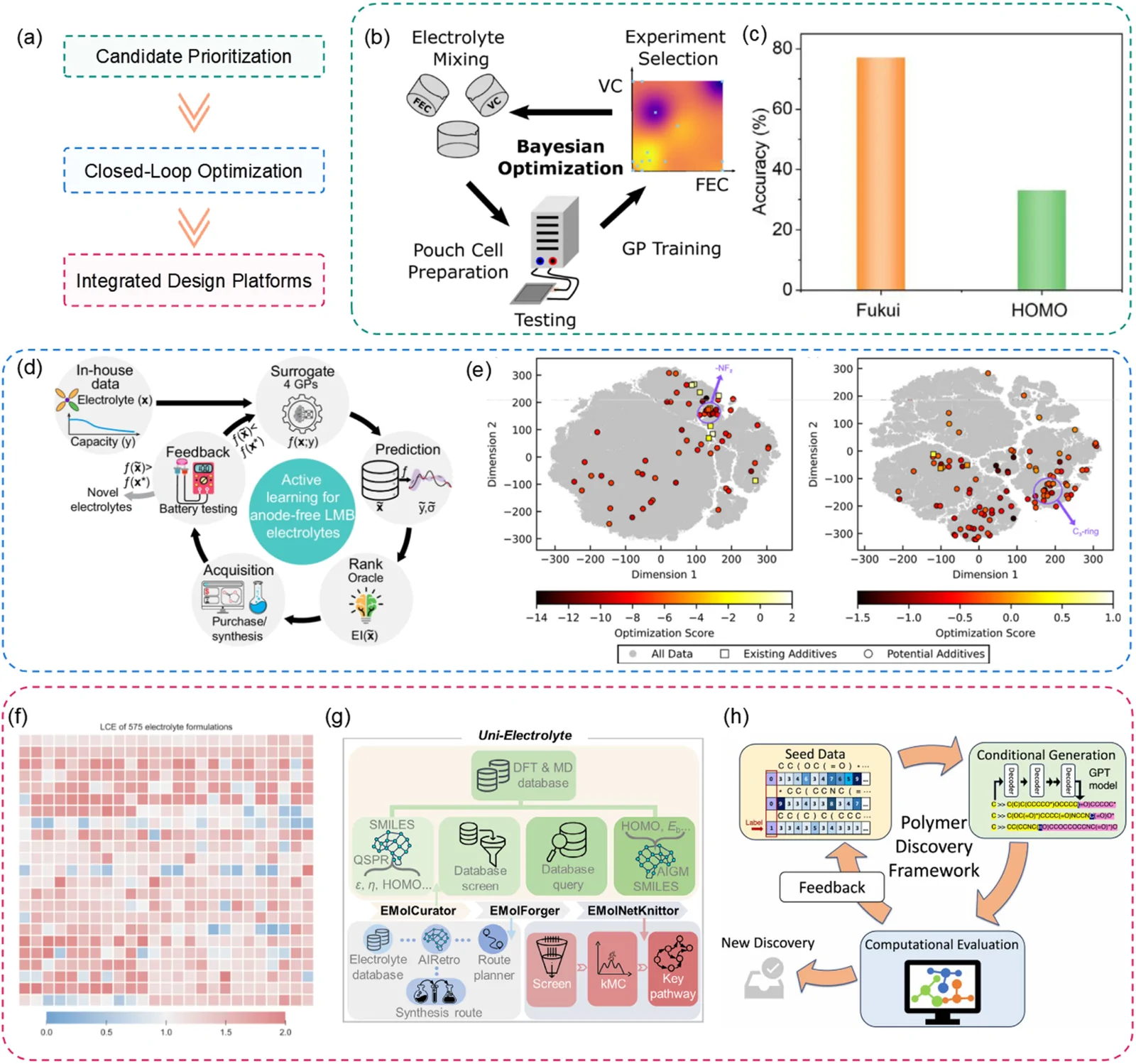
Accelerated screening and closed-loop optimization of molecular additives. (a) Schematic overview of the workflow from candidate prioritization to closed-loop optimization and integrated design platforms. (b) Bayesian optimization workflow tuning FEC/VC concentrations to maximize pouch-cell lifetime.^139^ Copyright 2022 Wiley. (c) Comparative analysis of Fukui-function-guided and HOMO-based additive screening methods, highlighting the improved predictive accuracy of reactivity-guided screening for high-temperature, high-voltage additives.^140^ Copyright 2025 Wiley. (d) Five-step active-learning workflow for optimizing anode-free lithium-metal-battery electrolytes under data-scarce conditions.^141^ Copyright 2025 Springer Nature. (e) Chemical-space mapping of Bayesian-optimized additives, highlighting SEI-forming difluoroamino motifs and cyclic leveling agents.^142^ Copyright 2024 American Chemical Society. (f) Heatmap of coulombic efficiency for electrolyte formulations generated by the automated AHTech robotic platform.^143^ Copyright 2025 American Association for the Advancement of Science. (g) Schematic representation of the Uni-Electrolyte platform, showing molecular curation, generation, retrosynthesis prediction, and SEI-network analysis.^144^ Copyright 2025 Wiley. (h) Iterative discovery framework based on conditional generative modeling, computational evaluation, and feedback-driven retraining for materials design.^145^ Copyright 2025 Royal Society of Chemistry.

Although predictive models and descriptor-based screening have improved additive selection, the practical search for effective formulations still faces a major bottleneck: multidimensional chemical spaces remain costly to explore and validate experimentally. Machine learning is therefore increasingly used not just to rank candidates, but to accelerate the full discovery cycle through prioritization, iterative updating, and platform-level design. From this perspective, the workflow progresses from candidate prioritization in defined additive spaces to closed-loop optimization under data constraints, and finally to integrated design platforms for additive discovery (Fig. 9a).

#### 4.2.1 Candidate prioritization in defined additive spaces

Moving beyond property prediction toward experimental optimization, Hildenbrand *et al.*^139^ applied Bayesian optimization to determine optimal additive concentrations in lithium-ion electrolytes. Using a Gaussian process surrogate model, the workflow iteratively refined formulations according to conductivity and stability feedback. This strategy efficiently navigated the compositional space of fluoroethylene carbonate and vinylene carbonate, significantly extending the cycle life of NMC622/graphite pouch cells (Fig. 9b).

At a more targeted screening stage, Kang *et al.*^140^ introduced a reactivity-guided screening strategy for electrolyte additives in high-temperature, high-voltage lithium batteries using DFT-derived Fukui functions rather than frontier-orbital descriptors alone. By evaluating local electrophilic and nucleophilic reactivity, they showed that additives with initial reaction sites on inorganic functional units are more likely to induce favorable interphase chemistry. Guided by this framework, 4-(trifluoromethyl)thiobenzamide was identified as a promising additive and experimentally verified to regulate Li^+^ solvation and promote a robust inorganic-rich interphase (Fig. 9c).

Overall, this first step of accelerated discovery is not yet a fully autonomous search. Instead, it focuses on shrinking the candidate pool early and prioritizing molecules that are more likely to yield useful interfacial chemistry before costly experimental validation begins.

#### 4.2.2 Closed-loop optimization under data constraints

To tackle optimization under severe data constraints, Ma *et al.*^141^ implemented an active learning strategy for anode-free lithium metal batteries. The workflow proceeds through model training, candidate ranking, synthesis evaluation, experimental validation, and iterative updating (Fig. 9d). Starting from a sparse in-house dataset, the model converged on four high-performance solvent systems within seven iterative campaigns, illustrating the efficiency of uncertainty-guided learning in data-scarce regimes.

Further refining adaptive search strategies, Lee *et al.*^142^ applied Bayesian optimization to identify dendrite-suppressing additives. Chemical-space mapping distinguished two key classes, namely SEI-forming species with difluoroamino motifs and cyclic leveling agents (Fig. 9e). These categories function synergistically to improve interfacial stability and guide more uniform lithium deposition. By integrating LUMO energy and donor number into the decision loop, the framework efficiently uncovered unconventional candidates that simultaneously optimized electrochemical stability and ionic conductivity.

Bridging the computational-experimental divide, Lin *et al.*^143^ developed an automated high-throughput electrochemical platform for aqueous zinc batteries. This robotic workflow integrates precision liquid handling, miniaturized electrodes, automated data acquisition, and real-time machine-learning analysis, enabling rapid evaluation of electrolyte formulations (Fig. 9f). Through large-scale screening, the platform identified effective additives such as *cis*-4-hydroxy-d-proline and clarified broader structure–property relationships across the tested space.

Taken together, these studies show that closed-loop optimization improves additive discovery not by replacing experiments, but by making each experimental round more informative. In this sense, the core advance lies in how the search space is continuously updated rather than exhaustively explored.

#### 4.2.3 Integrated design platforms for additive discovery

Beyond iterative optimization within predefined additive spaces, recent studies are beginning to integrate generation, screening, synthesis planning, and evaluation into more unified design platforms. Chen *et al.*^144^ developed Uni-Electrolyte, an integrated artificial intelligence platform for electrolyte molecule design that combines property prediction, multi-criterion screening, similarity search, generative design, retrosynthesis planning, and SEI-reaction analysis. Rather than treating molecular generation as an isolated task, this platform connects candidate proposal with synthetic accessibility and interphase chemistry, thereby extending electrolyte discovery from virtual screening to a more complete design workflow (Fig. 9g).

A related platform-oriented strategy was proposed by Khajeh *et al.*^145^ through an iterative framework based on conditional generative models, computational evaluation, and feedback-driven retraining. Using polymer electrolytes as a proof-of-concept system, they showed that generative models can move beyond one-shot proposal and progressively refine candidates through repeated evaluation cycles, ultimately yielding polymers with ionic conductivities exceeding the original training distribution and even surpassing polyethylene oxide benchmarks in molecular dynamics simulations (Fig. 9h). Although this work focuses on polymer electrolytes rather than small-molecule additives, it provides an instructive methodological template for additive discovery when generation, evaluation, and feedback need to be tightly coupled.

More broadly, this third layer extends additive discovery from accelerated selection to more integrated design. The key shift is that AI is no longer used only to rank existing options, but also to coordinate the full chain from candidate proposal to mechanistic evaluation.

### 4.3 Reasoning-guided and data-enabled additive discovery

**Fig. 10:**
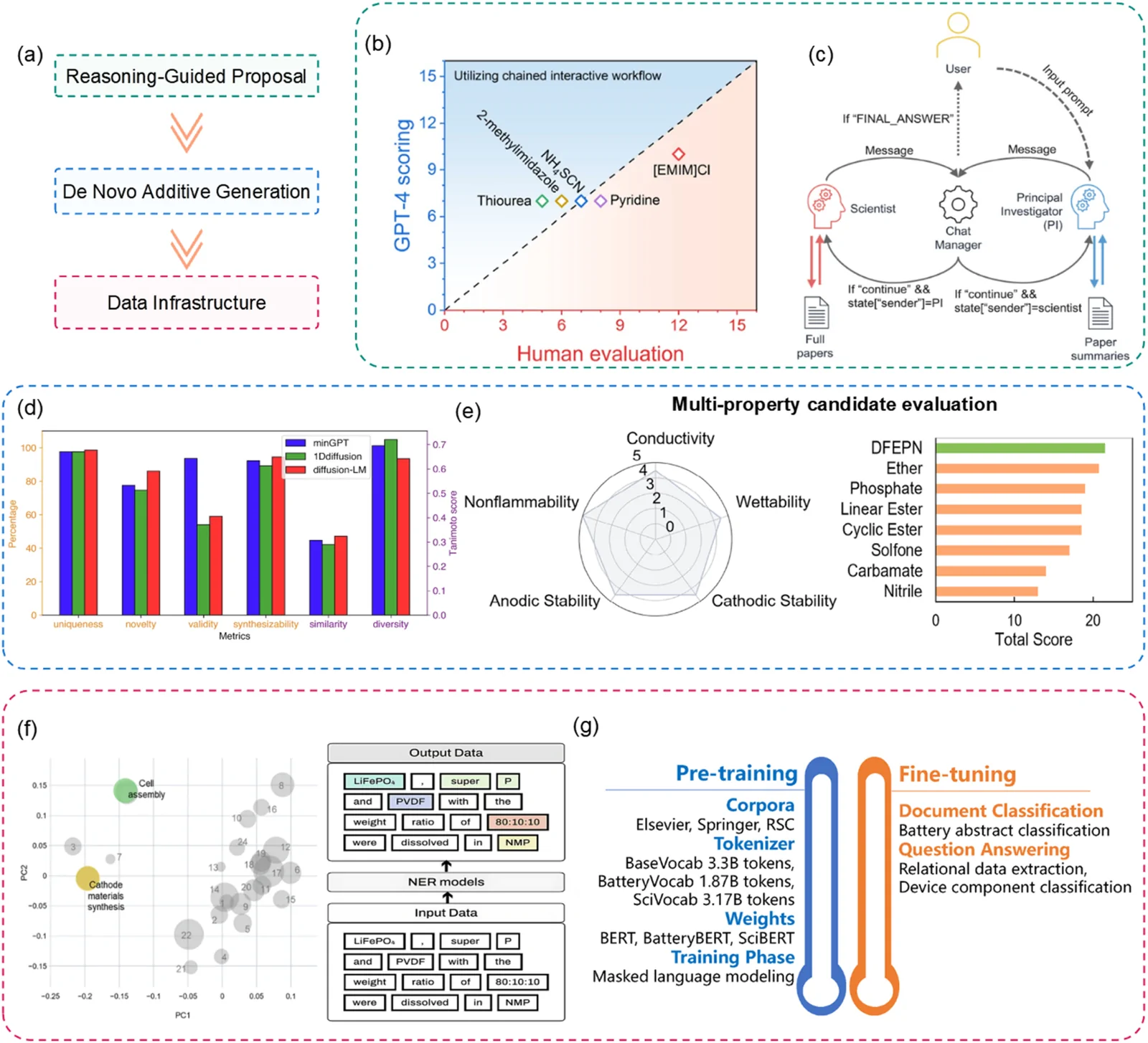
Reasoning-guided and data-enabled additive discovery. (a) Schematic overview of the workflow from reasoning-guided proposal to *de novo* additive generation and data infrastructure. (b) Scatter plot demonstrating the agreement between GPT-4 scoring and human evaluation for additive candidates.^146^ Copyright 2025 Science China Press. (c) Multi-agent reasoning workflow in which scientist and principal investigator agents collaborate for autonomous electrolyte design.^147^ Copyright 2025 Wiley. (d) Performance comparison of transformer- and diffusion-based generative models for *de novo* electrolyte design.^148^ Copyright 2024 Springer Nature. (e) Multi-property evaluation and prioritization of the generated additive DFEPN, highlighting conductivity, wettability, anodic/cathodic stability, and overall score as practical screening indicators for electrolyte additive candidates.^149^ Copyright 2025 Wiley. (f) Text-to-battery-recipe pipeline utilizing NLP to extract structured formulation data from unstructured battery literature.^150^ Copyright 2025 Springer Nature. (g) Two-stage workflow of BatteryBERT, showing battery-domain pretraining followed by fine-tuning for downstream tasks such as document classification and extractive question answering.^151^ Copyright 2022 American Chemical Society.

Although closed-loop optimization improves the efficiency of additive screening, discovery remains constrained by fragmented literature knowledge, sparse structured data, and the difficulty of moving beyond known molecular libraries. Artificial intelligence is now beginning to relieve this bottleneck by linking literature-derived rules, candidate generation, and data infrastructure within a more connected discovery workflow. In this progression, additive discovery advances from reasoning-guided proposal to *de novo* additive generation and finally to the data infrastructure needed for more autonomous design (Fig. 10a).

#### 4.3.1 Reasoning-guided proposal of additive candidates

To address the scarcity of structured mechanistic knowledge, the field is beginning to use large language models to elevate workflows from data processing to semantic reasoning and automated candidate proposal. This is particularly relevant for electrochemical interfaces, where high-fidelity descriptors often depend on computationally expensive simulations or large screening campaigns. Generative AI and LLMs help bridge this gap by extracting implicit design rules from fragmented literature and using them to guide candidate selection.

In low-data electrolyte systems, Chen *et al.*^146^ developed a human-AI framework for Zn–iodine electrolytes. A strong agreement between GPT-4 scores and expert evaluations confirmed the reliability of this iterative workflow (Fig. 10b). The protocol decomposes reasoning into discrete stages, including mechanism synthesis, design-principle formulation, and candidate generation, making it especially useful in low-data regimes.

Advancing human–machine collaboration, Robson *et al.*^147^ developed a retrieval-augmented multi-agent system for autonomous discovery. A principal investigator agent formulates the strategy while a scientist agent evaluates feasibility within the same workflow (Fig. 10c). Supported by vectorized literature databases, this architecture proposed novel Lewis base combinations that were later validated experimentally. The study demonstrates that retrieval-grounded agents can function as context-aware proposal systems, compressing the cycle from conceptual reasoning to empirical testing.

More importantly, this layer of discovery does not replace electrochemical validation, but it substantially improves how additive knowledge is organized and how promising candidates are proposed. Its main contribution is to transform diffuse literature knowledge into testable molecular hypotheses.

#### 4.3.2 *De novo* additive generation

Beyond proposing candidates from existing knowledge, recent studies are beginning to use generative models to directly create new electrolyte molecules. Yang *et al.*^148^ positioned generative models as frameworks for molecular design. Transformer- and diffusion-based approaches were shown to expand chemical space while maintaining high validity and synthesizability (Fig. 10d). Notably, a transformer model fine-tuned with molecular dynamics data generated novel structures with conductivities surpassing those of the training set. Although the study is framed more broadly around electrolyte design, its methodology is directly relevant to additive generation.

Moving from a transferable methodology to additive-specific implementation, Liu *et al.*^149^ developed a deep-learning-assisted generative model for the multiobjective design of lithium-metal-battery electrolyte additives. To address data scarcity, they expanded limited structure–performance data into a 70 095-point multiproperty dataset and coupled it with a graph generative framework capable of sampling structurally diverse molecules. This workflow produced a newly generated additive, denoted as DFEPN, that simultaneously improves flame resistance and electrochemical stability. Compared with the PFPN reference, DFEPN delivered better capacity retention and promoted more stable dual interphases, illustrating how generative models can move additive discovery from screening existing libraries toward targeted creation of multifunctional molecules (Fig. 10e). The multiproperty profile of DFEPN further illustrates that *de novo* generated additives require post-generation evaluation beyond a single predicted activity score, linking molecular generation to practical candidate prioritization.

In this sense, this subsection marks the clearest transition from selection to invention. The main advance is that additive discovery no longer needs to be restricted to known libraries, but can increasingly proceed through deliberate generation of molecules with multiple targeted functions.

#### 4.3.3 Data infrastructure for autonomous additive discovery

Autonomous additive discovery also depends on a reliable information backbone. Lee *et al.*^150^ engineered a text-to-battery-recipe pipeline that couples topic modeling with transformer-based named entity recognition to convert unstructured literature into structured datasets (Fig. 10f). This hybrid model accurately extracts solvent, salt, and additive parameters, significantly outperforming general few-shot prompting. By automating the compilation of critical metrics such as dosage and efficiency, such text-mining frameworks provide the large-scale curated datasets required for more robust machine-learning workflows.

At the language-model level, Huang and Cole developed BatteryBERT,^151^ a battery-domain pretrained language model designed to improve information retrieval and data extraction from the rapidly expanding battery literature. After pretraining on a large battery-paper corpus, the model was fine-tuned for downstream tasks including battery-paper classification and extractive question answering for device-component identification, and was further used to enhance an existing battery database. Relative to general BERT baselines, BatteryBERT improved performance on battery-specific text-mining tasks, demonstrating how domain-adapted language models can strengthen the textual infrastructure needed for scalable literature extraction and database enhancement in data-driven electrolyte and additive research (Fig. 10g).

In this context, data infrastructure should connect literature extraction, computational screening, experimental validation, and database curation through provenance-preserving records, so that hypotheses mined from the text can be cross-checked by calculations or experiments before being incorporated into high-confidence additive datasets.

Taken together, the practical value of machine learning in molecular additive discovery lies not only in accelerating prediction, but also in converting chemical and interfacial information into operational screening rules.^152^ For molecular additives, these rules should connect the molecular structure with specific interfacial functions, including coordination remodeling, interfacial adsorption, electrochemical stability, solubility, ion-transport compatibility, and competitive inhibition.^153^ In this context, autonomous discovery is not driven by generative models alone; it also requires domain-specific pipelines that organize data, descriptors, and models into structured knowledge for iterative design.^154^ Therefore, ML-guided additive discovery should be treated as a decision-making workflow rather than as a single prediction step, following four practical rules: grouping candidate molecules in chemical space to avoid redundant screening, selecting descriptors according to targeted interfacial functions, ranking candidates under multiple practical constraints, including electrochemical stability, solubility, ionic-transport compatibility, interfacial affinity, safety or nonflammability, and synthetic accessibility, and using uncertain or failed candidates as feedback for the next screening cycle.^155^

In practical ML-guided additive discovery, clustering and screening can first be used to narrow chemical space according to interface-related descriptors. These descriptors may include solvation-related features, redox stability, adsorption energy, interfacial affinity, ion-transport compatibility, surface coverage, and inhibition-related features. Additives selected in this way can then be considered for targeted interface modification, such as regulating solvation structures, stabilizing interphases or catalytic surface states, suppressing parasitic reactions, tuning adsorbate binding, or inhibiting competing pathways. These design rules, however, should not be interpreted as a substitute for realistic interfacial validation. Molecular additives operate at electrified and dynamically evolving interfaces, where electrode potential, explicit solvent and ion distributions, interfacial electric fields, local solvation structures, surface coverage, and reaction kinetics jointly determine performance. Recent ML-assisted electrochemical-interface simulations also emphasize that reliable prediction requires physically meaningful interface models rather than descriptor-based ranking alone.^156^ Explicit-solvation studies at liquid-solid interfaces further demonstrate that the solvent structure can strongly affect catalytic activity, selectivity, and reaction pathways, making solvent and ion environments essential variables in additive evaluation.^157^ Interface-specific datasets, including AI-accelerated AIMD/MLMD datasets for electrochemical interfaces, highlight the need for curated trajectories, standardized metadata, and cross-study comparability.^158^ In addition, machine-learning potentials that incorporate interfacial dielectric response emphasize that realistic electrochemical modeling should account for both electrolyte polarization and electrode charge response.^159^ Therefore, practical ML-guided additive discovery should follow a tiered validation strategy: low-cost descriptors and clustering can be used for early chemical-space reduction, whereas high-priority candidates should be further examined using explicit-solvent simulations, electrified or constant-potential interface models, AIMD/MLMD or reactive simulations, and *in situ*/*operando* evidence whenever possible.

## 5 Conclusion and future perspectives

### 5.1 Current limitations and boundary conditions

Despite the demonstrated interfacial leverage of molecular additives, the transition from empirical discovery to predictive design remains constrained by structural bottlenecks in data governance and physical fidelity. The dominant limitations differ from simple model sophistication; rather, they stem from three foundational gaps regarding interfacial realism, representational generalization, and evaluation rigor.

#### 5.1.1 Lack of interfacial realism and data interoperability

While experimental and computational data volumes are expanding, the field lacks interoperable datasets that capture realistic interfacial conditions. In batteries, additive efficacy is path-dependent and governed by electrolyte backbones, impurity levels, and interphase history, yet such metadata are frequently omitted in device-level reports.^160^ Similarly, in electrocatalysis, critical drivers like local pH and potential-driven reconstruction are rarely curated systematically. Although simulations offer scale, they often idealize interfaces, leading to a disconnect where computational proxies fail to align with the complex boundary conditions of physical experiments.^161–163^ Without standardized reporting that bridges these gaps, large-scale datasets risk amplifying noise rather than clarifying transferability.^164,165^

#### 5.1.2 The trade-off between generality and contextual fidelity

A central tension exists between endpoint-first screening and mechanism-anchored design. Many current models map intrinsic molecular features directly to device endpoints (*e.g.*, efficiency or lifetime). While scalable, this approach often fails under domain shifts because it bypasses the governing intermediates, namely solvation, adsorption, and film formation.^166^ This creates a rigorous “boundary of validity”: static proxies can function as initial filters but risk systematic misranking when formulation chemistries or operating windows change.^31,167–169^ Addressing this requires a shift toward context-aware representations that balance broad chemical coverage with sufficient environmental inputs to capture specific interfacial interactions.^170^

#### 5.1.3 Non-standard evaluation and the illusion of generalization

Clarifying boundary conditions is not merely a technical detail but a prerequisite for defining when AI models are valid.^171,172^ Current evaluation practices often overestimate reliability, as random splits on homogeneous datasets can produce high scores that collapse when applied to new electrolyte systems or catalysts. True predictive value hinges on robustness under domain shifts, including transfers across disparate backbones or protocols, yet such stress tests are not routine. For the shared-primitives framework proposed in this review, this issue is particularly important because descriptor transferability cannot be inferred from conceptual mapping alone. Future benchmarks should compare primitive-anchored descriptors with system-specific descriptors under identical validation protocols, using leave-one-system-out tests, cross-chemistry prediction errors, descriptor-ablation analysis, in-domain/out-of-domain performance drops, and calibrated uncertainty estimates. Furthermore, the lack of rigorous uncertainty quantification limits the ability to flag unreliable extrapolations.^173,174^ To move beyond proof-of-concept demonstrations, the community must adopt domain-shift-aware benchmarks that rigorously distinguish between memorizing training data and learning transferable physical rules.^175^

### 5.2 Application prospects

In response to the limitations outlined above, the next phase of data-driven additive research must transition from fragmented trial-and-error to reusable, mechanism-informed ecosystems. By prioritizing interfacial realism and physics-grounded interpretability, we can construct the “self-driving” workflows necessary for next-generation interfaces.

#### 5.2.1 End-to-end closed loops and staged workflows

Future frameworks will integrate computational and experimental pipelines into unified design-test-update cycles.^176^ A representative demonstration is the robotic Bayesian optimization workflow reported by Dave *et al.*, which autonomously optimized non-aqueous electrolytes under complex constraints.^177^ To scale this logic, we envision “staged workflows” where rapid computational prescreening filters vast chemical spaces, followed by targeted high-fidelity experimental validation. This structure ensures that expensive closed-loop resources are focused on candidates with high physical feasibility. By explicitly setting our proposed mechanistic primitives (*P*1–*P*3), such as specific solvation energies or radical scavenging rates, as intermediate reward functions, this approach effectively bridges the gap between high-throughput screening and deep mechanistic verification.

#### 5.2.2 Physics-interpretable composite descriptors

Transferable design rules require moving beyond “black-box” molecular features toward “minimally sufficient context,” defined as representations that are complex enough to capture interfacial physics but simple enough to generalize. As illustrated by Weng *et al.* using symbolic regression, compact and interpretable descriptors can guide experimentally verifiable leads by linking the structure explicitly to intermediate targets.^178^ In this view, future models will likely predict hierarchical proxies, such as solvation energy (*P*1) or film-forming potential (*P*2), as stepping stones to device-level performance. This ensures that AI learns physical causation rather than spurious correlation.

#### 5.2.3 Uncertainty-aware optimization and robust evaluation

To make decision-making traceable in constraint-rich spaces, active learning must explicitly quantify what it does not know. Rohr *et al.* quantified the efficiency gains achievable with sequential learning, reporting up to ∼20× speedup by balancing exploration (uncertainty) and exploitation (performance).^179^ However, true utility hinges on rigorous evaluation. Future optimization platforms must adopt domain-shift-aware diagnostics to verify that reported speedups are real acceleration rather than artifacts of a narrow training domain. Standardizing these uncertainty metrics is the final step to move from “demonstration-grade” algorithms to “deployment-grade” discovery tools. Ultimately, anchoring these autonomous platforms in unified mechanistic primitives (*P*1–*P*3) will efficiently translate complex interfacial phenomena into predictive, data-driven design pathways for next-generation electrochemistry.

## Author contributions

Guanyu Wang: conceptualization, investigation, data curation, visualization, writing – original draft, and writing – review & editing. Shun Zou, Siyuan Lai, Shujie Zhang, Yumo Zhang, Bowen Jiang, and Yujie Shi: investigation, visualization, writing – original draft (selected sections), and writing – review & editing. Guobin Wen, Hongshuai Hou, and Xiaobo Ji: supervision and writing – review & editing. Bohua Ren: conceptualization, supervision, project administration, funding acquisition, and writing – review & editing. All authors reviewed and approved the final manuscript.

## Conflicts of interest

There are no conflicts to declare.

## Data Availability

No primary research results, software or code have been included, and no new data were generated or analysed as part of this review.
